# Beyond Classical Reward Circuits: Dorsal Striatum Proteomic Alterations in an Adolescent Phencyclidine-Induced Mouse Model of Schizophrenia and Nicotine Use

**DOI:** 10.3390/ijms27209328

**Published:** 2026-10-20

**Authors:** Thainá Pereira Souza, Andrés Rodríguez-Vega, Ana Carolina Dutra-Tavares, Keila Alves Semeão, Claudio Carneiro Filgueiras, Anderson Ribeiro-Carvalho, Alex Christian Manhães, Yael Abreu-Villaça

**Affiliations:** 1 Laboratório de Neurofisiologia, Departamento de Ciências Fisiológicas, Instituto de Biologia Roberto Alcantara Gomes, Universidade do Estado do Rio de Janeiro (UERJ), Av. Prof. Manuel de Abreu 444, 5 Andar—Vila Isabel, Rio de Janeiro 20550-170, RJ, Brazil; thainasouza2@gmail.com (T.P.S.); andresve22@gmail.com (A.R.-V.); brainherkeila@gmail.com (K.A.S.); ccfilg@yahoo.com.br (C.C.F.); ac_manhaes@yahoo.com.br (A.C.M.); 2 Departamento de Ciências Biomédicas e Saúde, Instituto de Biologia Roberto Alcantara Gomes, Universidade do Estado do Rio de Janeiro (UERJ), Cabo Frio 28905-320, RJ, Brazil; dutratavaresana@gmail.com; 3 Departamento de Ciências, Faculdade de Formação de Professores, Universidade do Estado do Rio de Janeiro (UERJ), São Gonçalo 24435-005, RJ, Brazil; ribeiro_carvalho@yahoo.com.br

**Keywords:** mental disorder, nicotine addiction, proteome, e-cigarette, mass spectrometry, prodrome, co-exposure, basal ganglia, GAPDH, FKBP1A

## Abstract

Schizophrenia and nicotine misuse comorbidity frequently emerges during adolescence. Although the dorsal striatum has received less attention than mesolimbic regions in this context, growing evidence supports its involvement in both schizophrenia-related dysfunction and the transition to compulsive drug-seeking. Here, we investigated dorsal striatal proteomic alterations in adolescent male and female C57BL/6 mice modeled to schizophrenia (phencyclidine: 2.5 mg/kg, s.c., 15 days) with a history of nicotine exposure (nicotine: 24 mg/kg/day, minipump infusions, 7 days). Dorsal striatum samples were analyzed by label-free quantitative proteomics, followed by Reactome pathway enrichment and interactome map analyses. Co-exposure induced sex-dependent alterations. Females exhibited greater proteomic disruption, whereas in males, co-exposure amplified phencyclidine-associated alterations while attenuating nicotine-driven effects. Across sexes, convergent enrichment was observed in pathways related to immune signaling, metabolic regulation and signal transduction. Female interactome analysis identified glyceraldehyde-3-phosphate dehydrogenase (Gapdh) as the most highly connected node linked to proteins involved in bioenergetics, RNA processing, and apoptosis, while the male network was centered on peptidyl-prolyl cis-trans isomerase (Fkbp1a) and associated with proteostasis-related processes. Our results identify the dorsal striatum as an emerging molecular target in an animal model combining schizophrenia-related and nicotine exposure-related insults and highlight pathways and candidate molecular markers to be further investigated and validated.

## 1. Introduction

Tobacco misuse and SCHZ are closely intertwined psychiatric conditions. This comorbidity is highly prevalent, exceeding by 2–4× the rates observed in individuals without mental disorders [1,2]. It also emerges early, typically during adolescence, when pre-psychotic, cognitive, and negative symptoms of SCHZ first appear, coinciding with experimentation and progression to the misuse of tobacco products [3,4,5]. Accordingly, the prevalence of tobacco use in the prediagnostic prodromal phase of SCHZ ranges from 17% to 46% [6] and rises to between 59% and 78% among patients experiencing a first episode of psychosis [7,8].

Despite the recognition of the period of adolescence as critical for SCHZ and tobacco misuse comorbidity, the early mechanisms linking these disorders remain incompletely understood. Nicotine exposure during adolescence has been associated with improvement of positive and negative-like symptoms of mice modeled to SCHZ [9,10], yet paradoxically, the proteome imbalance in the prefrontal cortex and nucleus accumbens is more severe in mice co-modeled to SCHZ and nicotine use than those only exposed to nicotine [11,12]. There are still few studies addressing the impact of consumption of tobacco products during the early course of SCHZ. First episode psychosis patients that are tobacco smokers may have higher levels of illness severity, higher psychiatric symptoms and lower quality of life relative to non-smokers [13,14]. However, cigarette smoking has also been associated with lower severity of depressive and negative symptoms [15], and nicotine vapers have a higher role and social functioning and lower negative symptom severity than non-vapers [16]. Recent onset SCHZ patients that smoke cigarettes were further shown to outperform non-smokers with the disorder on attentional measures [17].

Neurobiological maturation events may underlie adolescent susceptibility to both SCHZ and nicotine [18,19,20]. Several brain regions are only considered fully mature at adulthood. Particularly, the striatum, a subcortical structure that supports functions ranging from basic motor control to higher-order processes such as reward processing, acquisition of behavioral habits, learning cognitive skills and emotion-related patterns of action [21], continues to undergo substantial maturation during adolescence [22,23]. The mapping of striatal connections has shown partially segregated pathways consistent with the segmentation of the striatum into limbic, associative and sensorimotor parts (for review: [24]). The limbic striatum, also known as ventral striatum, comprises the nucleus accumbens. Regarding the associative striatum, it includes the caudate and rostral putamen, while the caudal putamen composes the sensorimotor striatum (for review: [24]). The associative and sensorimotor parts are also known as dorsal striatum.

There is strong evidence linking a dysfunctional striatum to SCHZ, as an excess of striatal dopamine has long been associated with the positive symptomatology of the disorder [25]. In this context, drug-naive SCHZ patients exhibit elevated dopamine presynaptic storage capacity as well as baseline and amphetamine-evoked intrasynaptic release in the striatum when compared to control individuals, and those events associate with the emergence or worsening of positive psychotic symptoms [26,27]. Despite these findings, the precise locus of striatal dysfunction in SCHZ remains elusive. Initial clinical findings [28,29,30] pointed to the involvement of the nucleus accumbens in the expression of SCHZ-positive symptoms. Together with the known role of the dorsal striatum in motor functions [31], this led both preclinically and clinically to research efforts that focus on the investigation of mesolimbic pathways and mechanisms involved. However, advances in neurochemical imaging studies have since provided evidence that the dorsal striatum plays a central role in the pathophysiology of SCHZ [32]. Notably, the analysis of striatal dopaminergic function in individuals at clinical high risk (CHR) for psychosis shows a sensitized response to stress in its dorsal division (both associative and sensorimotor). Moreover, transition from the CHR state to overt psychosis is associated with elevated dopamine synthesis capacity in the dorsal, primarily sensorimotor, striatum, with no significant changes detected in the limbic region [33]. More recently, a meta-analysis that investigated the effect size of the dysregulation across striatum subdivisions identified increased presynaptic dopamine functioning in SCHZ patients compared to controls in the dorsal division, whereas this effect did not reach significance in the limbic subdivision [32].

When considering mechanisms that lead to substance use disorders, there are established roles for the ventral striatum that include the reinforcement of rewarding behaviors, the regulation of motivation, and decision-making [34,35]. In turn, studies suggest that drug-seeking habit formation and compulsion involve the progressive recruitment of the dorsal striatum [36]. Unlike its ventral division, the dorsal part of the striatum shows minimal involvement in the acute reinforcing properties of drugs of abuse but becomes increasingly engaged as drug use progresses to a chronic pattern [36,37]. These data raise the possibility that circuits that involve the dorsal striatum in part mediate mechanisms that underlie nicotine effects on SCHZ symptomatology. In this regard, while a handful of studies that focused on adolescence describe the involvement of mesolimbic regions [12,38,39], the relevance of the dorsal striatum has not been investigated.

Animal models stand out as translationally relevant for the study of effects and mechanisms of action of both nicotine and SCHZ. Phencyclidine, a non-competitive antagonist of N-methyl-D-aspartate (NMDA) receptors, is widely used to induce SCHZ-like behaviors in rodents [40], deficits in sensorimotor gating [41], and neurochemical alterations [42] analogous to those observed in SCHZ. Our group has employed phencyclidine exposure in mice to reproduce key features of SCHZ during adolescence and early adulthood [9,10,11,12,43]. In parallel, osmotic minipumps, a low-stress method that allows continuous drug delivery with precise control over dose and exposure duration [44,45], have been extensively used to expose rodents to nicotine [46]. In adolescent mice, this experimental strategy provides a suitable experimental framework for investigating nicotine–SCHZ combined insults [9,10,11,12]. Particularly, using the same experimental protocol of exposures adopted in the current study, our group was able to model positive, negative, and cognitive symptoms of SCHZ as well as sensorimotor gating deficits in adolescent mice exposed to phencyclidine. Nicotine history during adolescence interfered with these results, mitigating positive-like and negative-like behaviors elicited by phencyclidine, whereas memory and sensorimotor gating deficits were not affected [9].

Building on these findings, we used the same experimental models to perform label-free quantitative proteomics, followed by Reactome pathway enrichment and interactome mapping, focusing on the dorsal striatum, given its established roles in both SCHZ [32] and addictive behaviors [36,37]. Male and female animals were examined independently considering well-documented sex differences in SCHZ [47,48] and in nicotine-related outcomes [11,12]. Adolescent mice received subchronic phencyclidine treatment together with a brief period of nicotine exposure. We hypothesized that molecular profiling of the dorsal striatum would contribute to the identification of altered biological pathways and candidate molecular markers associated with the combined SCHZ- and nicotine-related insults.

## 2. Results

A total of 4261 unique peptides that corresponded to 320 proteins were identified using a minimum of two peptides per protein and that were present in at least two of the replicate analyses. The number of proteins was comparable to that identified in previous papers of our group that used a similar analytical approach [11,12]. In Supplementary Material File S1, Sheets S1 and S2 show a list of peptides containing identifiers for all dorsal striatum proteins. These proteins were also ranked based on their abundance relative to their total independent spectral counts.

Studies in SCHZ patients show sex differences in SCHZ prevalence, symptom domains, and severity, and to some extent, these differences are replicated in animal models [47,48]. Accordingly, here, mice were *a priori* separated by sex for the analyses, and the effects observed in females and males were not statistically compared. Most proteins (73,6%) were differentially expressed in only one sex (Supplementary Material File S1, Sheet S3). Also, for both males and females, changes in protein abundance depended on the drugs mice were exposed to, which corroborates the importance of considering phencyclidine and nicotine as independent factors in the analysis.

### 2.1. Proteomic Profile of the Dorsal Striatum of Mice Modeled to SCHZ, Nicotine Use, and the Comorbidity When Compared to Controls

**Figure 1:**
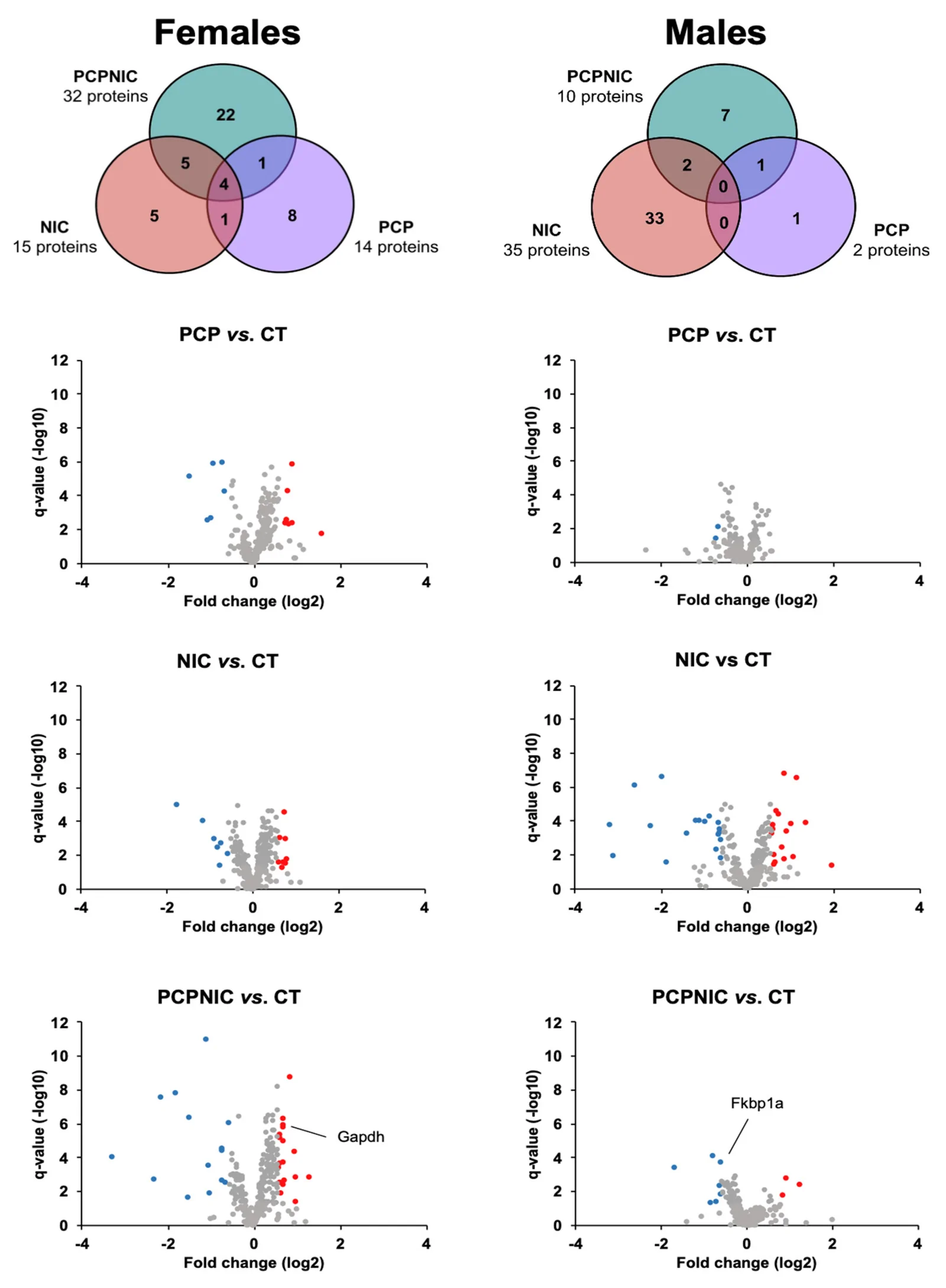
Venn diagrams (**top panels**) showing differentially abundant proteins in the dorsal striatum of female (**left**) and male (**right**) mouse models of schizophrenia (PCP), nicotine use (NIC), or the comorbidity (PCPNIC) when compared to controls (CT). Volcano plots (**lower panels**) provide a general picture of fold change magnitude (x−axis) and adjusted q-values (y−axis) of the dataset. Blue dots indicate downregulated proteins, red dots indicate upregulated ones, and gray dots indicate proteins that did not reach deregulation criteria (>1.5-fold change and adjusted q < 0.05). Candidate molecular nodes of the comorbidity are identified and will be discussed below.

The results from comparisons between CT and the other experimental groups (PCP, NIC and PCPNIC) are shown as Venn diagrams (Figure 1). In both PCPNIC and PCP groups, females exhibited a higher number of proteins that were differentially expressed than males. In the PCPNIC group, 32 proteins were differentially expressed in females (14 downregulated, 18 upregulated), compared with 10 in males (seven downregulated, three upregulated). In the PCP group, females had 14 differentially expressed proteins (six downregulated, eight upregulated), while PCP males had two (two downregulated, zero upregulated). Regarding the NIC group, males were more extensively affected, as there were 35 differentially expressed proteins in males (18 downregulated, 17 upregulated) and 15 in females (seven downregulated, eight upregulated). In Supplementary Material File S1, Sheets S4–S9 provide detailed information regarding the aforementioned proteins.

### 2.2. Exclusively Differentially Abundant Proteins in Mice Modeled to the Comorbidity

Exclusive proteins of the PCPNIC group are those differentially expressed only in this experimental group (Figure 1). The analysis of PCPNIC mice identified 22 exclusively differentially abundant proteins in females (eight downregulated, 14 upregulated) and seven proteins in males (four downregulated, three upregulated). Detailed information on the differentially abundant proteins involved in each comparison can be found in Supplementary Material File S1—Sheets S10 and S11.

**Figure 2:**
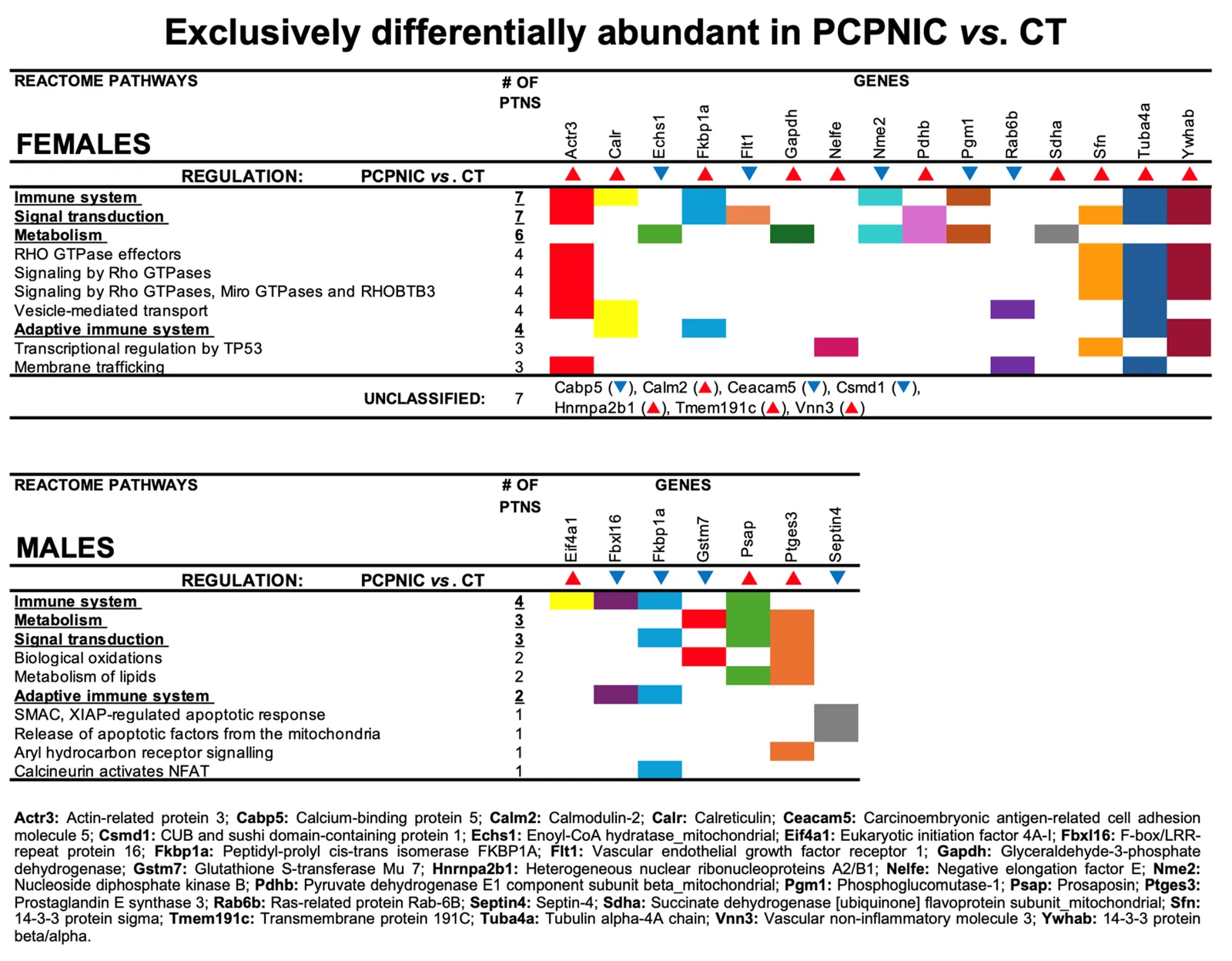
Data from exclusively differentially abundant proteins in the dorsal striatum of female and male mouse models of the comorbidity (PCPNIC) when compared to controls (CT). The panels show top 10 Reactome pathways. Pathways in bold and underlined were shared between sexes. ▼ indicates that a given protein is downregulated in PCPNIC mice when compared to CT ones, while ▲ indicates that a given protein is upregulated. #OF PTNS, number of proteins.

Among the 10 most enriched Reactome pathways identified in PCPNIC mice, four (immune system, adaptive immune system, signal transduction, and metabolism) were affected in both males and females (Figure 2). Pathways related to signaling by Rho GTPases, vesicle-mediated transport, membrane trafficking and transcriptional regulation by Tumor protein 53 (TP53, also known as Cellular tumor antigen p53) were present among the other top Reactome pathways only in females (Figure 2 upper panel). As for males, specific metabolism-related pathways (biological oxidations, metabolism of lipids) and pathways involved in apoptotic responses were enriched (Figure 2 lower panel). In Supplementary Material File S2, Figure S1 provides the complete list of Reactome pathways for each sex.

### 2.3. Commonly Differentially Abundant Proteins

The Reactome pathways analyses of proteins commonly differentially abundant for different comparisons are described below. These include analyses of proteins commonly differentially expressed in mice modeled to SCHZ and the comorbidity (PCP vs. CT and PCPNIC vs. CT), in mice modeled to nicotine use and the comorbidity (NIC vs. CT and PCPNIC vs. CT), and proteins commonly differentially expressed across SCHZ, nicotine use, and the comorbidity models (all groups vs. CT).

Because some comparisons described below yielded a limited number of differentially expressed proteins, the corresponding Reactome enrichment analyses were, in some instances, based on only one or a few input proteins. Therefore, these enrichment results should be interpreted as descriptive of potential biological processes associated with the observed proteomic changes rather than as definitive evidence of pathway dysregulation.

#### 2.3.1. Commonly Differentially Abundant Proteins in Mice Modeled to SCHZ and the Comorbidity (PCP vs. CT and PCPNIC vs. CT)

**Figure 3:**
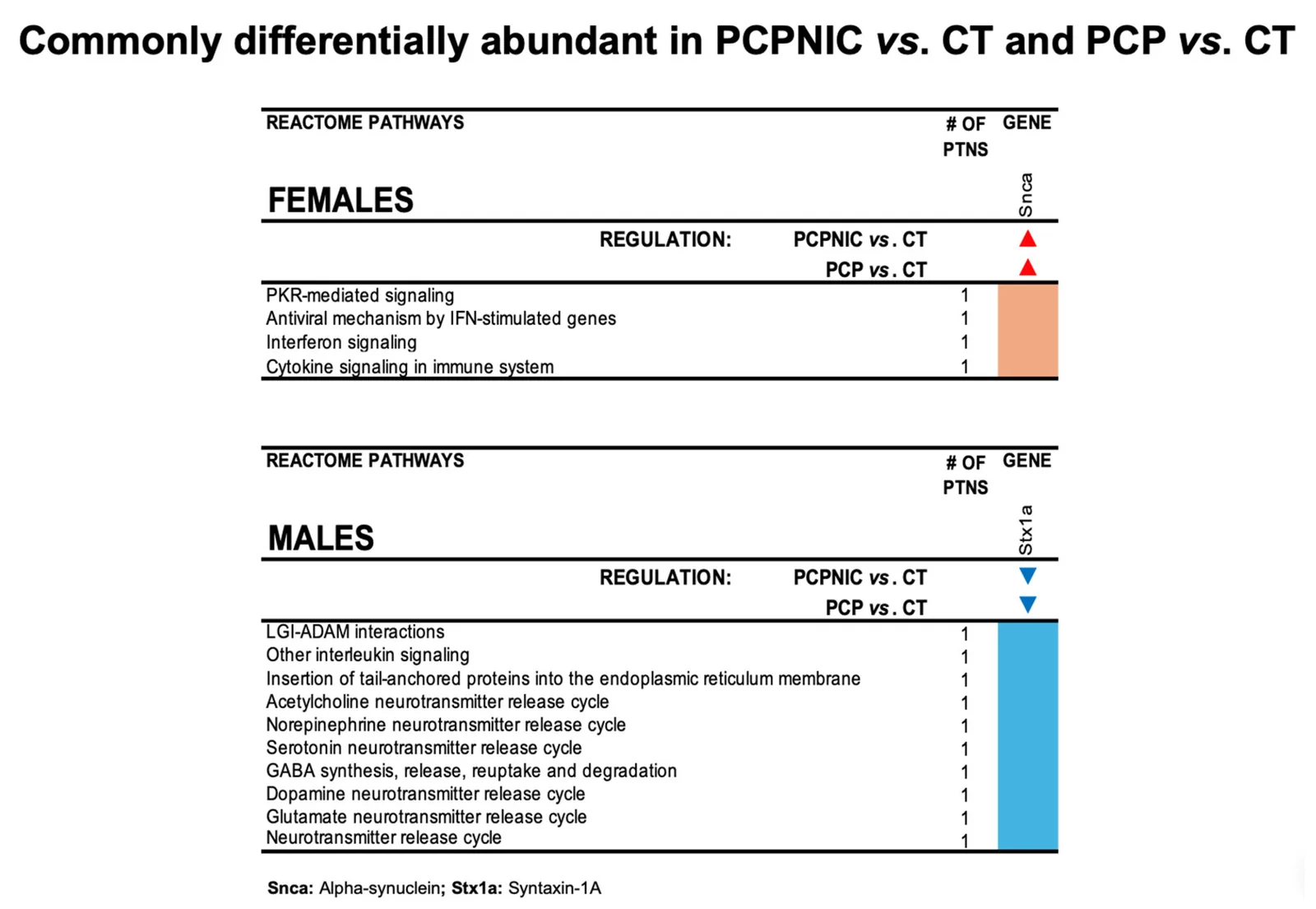
Data from proteins commonly differentially abundant in the dorsal striatum of female and male mouse models of schizophrenia (PCP) and the comorbidity (PCPNIC) when compared to controls (CT). The panels show top 10 Reactome pathways. ▼ indicates that a given protein is downregulated in PCP and PCPNIC mice when compared to CT ones, while ▲ indicates that a given protein is upregulated. # OF PTNS, number of proteins.

Commonly differentially abundant proteins in the dorsal striatum of PCP and PCPNIC mice represent the contributions of phencyclidine exposure to the profile of PCPNIC differentially expressed proteins (Supplementary Material File S1—Sheets S16 and S17). This analysis identified one upregulated protein in females and one downregulated protein in males (Figure 1). In females (Figure 3 upper panel), all four identified pathways were related to the immune system. As for males (Figure 3 lower panel), most of the 10 top pathways are involved with the release cycle of classic neurotransmitters, whereas one pathway was related to the immune system (other interleukin signaling). In Supplementary Material File S2, Figure S2 provides the complete list of Reactome pathways for females and males.

#### 2.3.2. Commonly Differentially Abundant Proteins in Mice Modeled to Nicotine Use and the Comorbidity (NIC vs. CT and PCPNIC vs. Ct)

**Figure 4:**
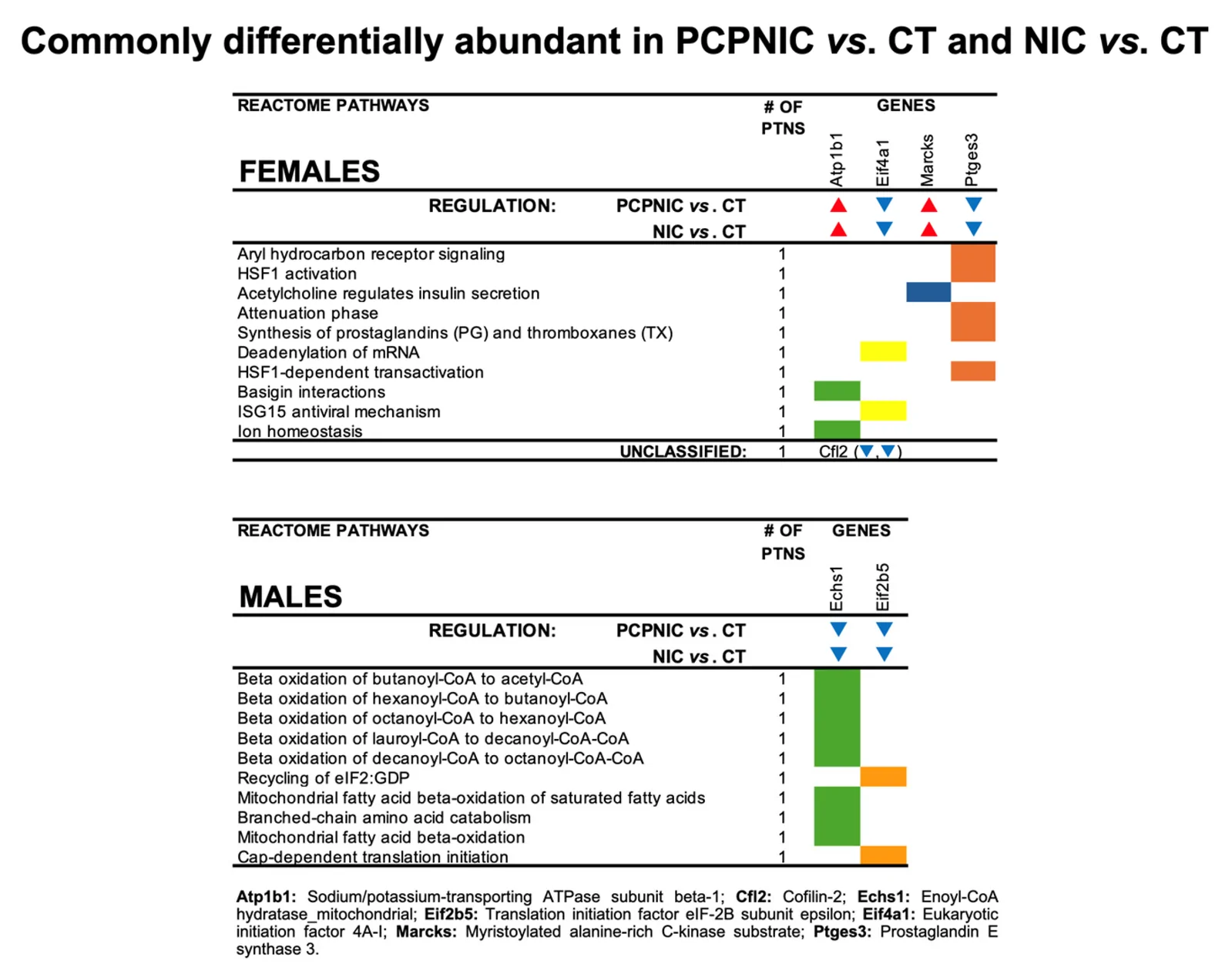
Data from proteins commonly differentially abundant in the dorsal striatum of female and male mouse models of nicotine use (NIC) and the comorbidity (PCPNIC) when compared to controls (CT). The panels show top 10 Reactome pathways. ▼ indicates that a given protein is downregulated in NIC and PCPNIC mice when compared to CT ones, while ▲ indicates that a given protein is upregulated. # OF PTNS, number of proteins.

The specific contribution of nicotine history to the dorsal striatal proteome of PCPNIC mice included (Figure 1), for females, five proteins (three downregulated, two upregulated) that were commonly differentially expressed in NIC and PCPNIC when compared to CT mice (Supplementary Material File S1—Sheet S18). As for males, two commonly downregulated proteins were identified (Supplementary Material File S1—Sheet S19). The Reactome analyses indicated that none of the 10 more frequent pathways were shared between females and males (Figure 4). In females, HSF1 activation-related processes and pathways involved with the immune and inflammatory responses and with the cell response to environmental and endogenous compounds emerged in the enrichment analysis (Figure 4 upper panel). The analysis of males indicated that most of the 10 top pathways are steps of fatty acid beta oxidation, with additional enrichment of pathways involved in translation initiation and leucine, isoleucine, and valine catabolism (Figure 4 lower panel). In Supplementary Material File S2, Figure S3 provides the complete list of Reactome pathways for each sex.

#### 2.3.3. Commonly Differentially Abundant Proteins in Mice Modeled to Nicotine Use, SCHZ and the Comorbidity (All Groups vs. CT)

**Figure 5:**
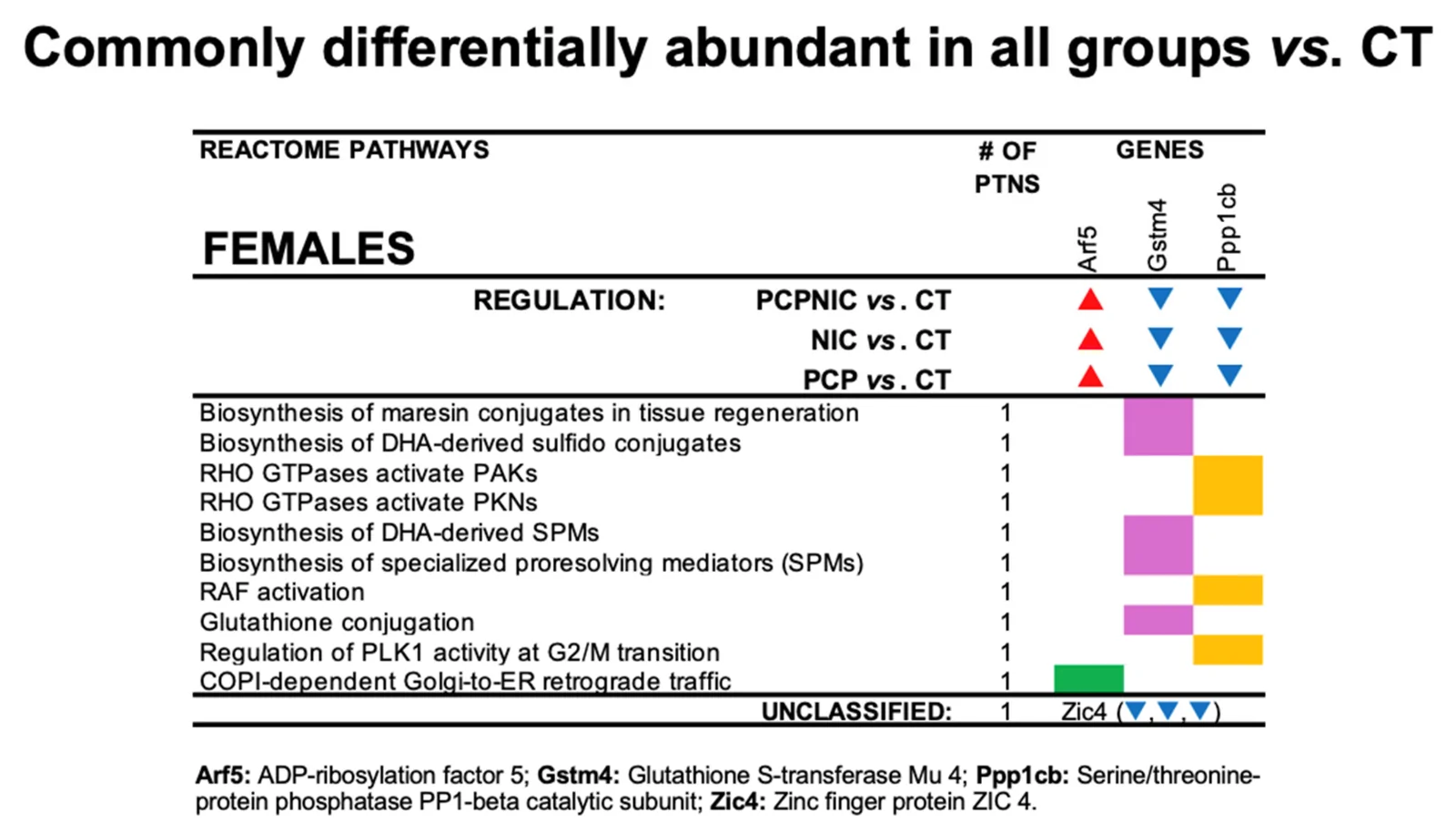
Data from proteins commonly differentially abundant in the dorsal striatum of female mouse models of schizophrenia (PCP), nicotine use (NIC), and the comorbidity (PCPNIC) when compared to controls (CT). The panel shows top 10 Reactome pathways. ▼ indicates that a given protein is downregulated in NIC, PCP, and PCPNIC mice when compared to CT ones, while ▲ indicates that a given protein is upregulated. # OF PTNS, number of proteins.

Proteins differentially abundant in all three drug-exposed groups indicate common contributions of PCP and NIC to the PCPNIC profile (Figure 1). In females, there were four differentially expressed proteins (three downregulated, one upregulated; Supplementary Material File S1—Sheet S20). Among the top 10 enriched Reactome pathways, several involved in the resolution of inflammation were identified in the enrichment analysis. Pathways associated with Rho-mediated cytoskeletal rearrangements, cell survival and proliferation also stood out (Figure 5). There were no commonly differentially expressed proteins in males. In Supplementary Material File S2, Figure S4 provides the complete list of Reactome pathways for females.

### 2.4. Interactome Maps of Differentially Abundant Proteins in the Dorsal Striatum of Mice Modeled to the Comorbidity

**Figure 6:**
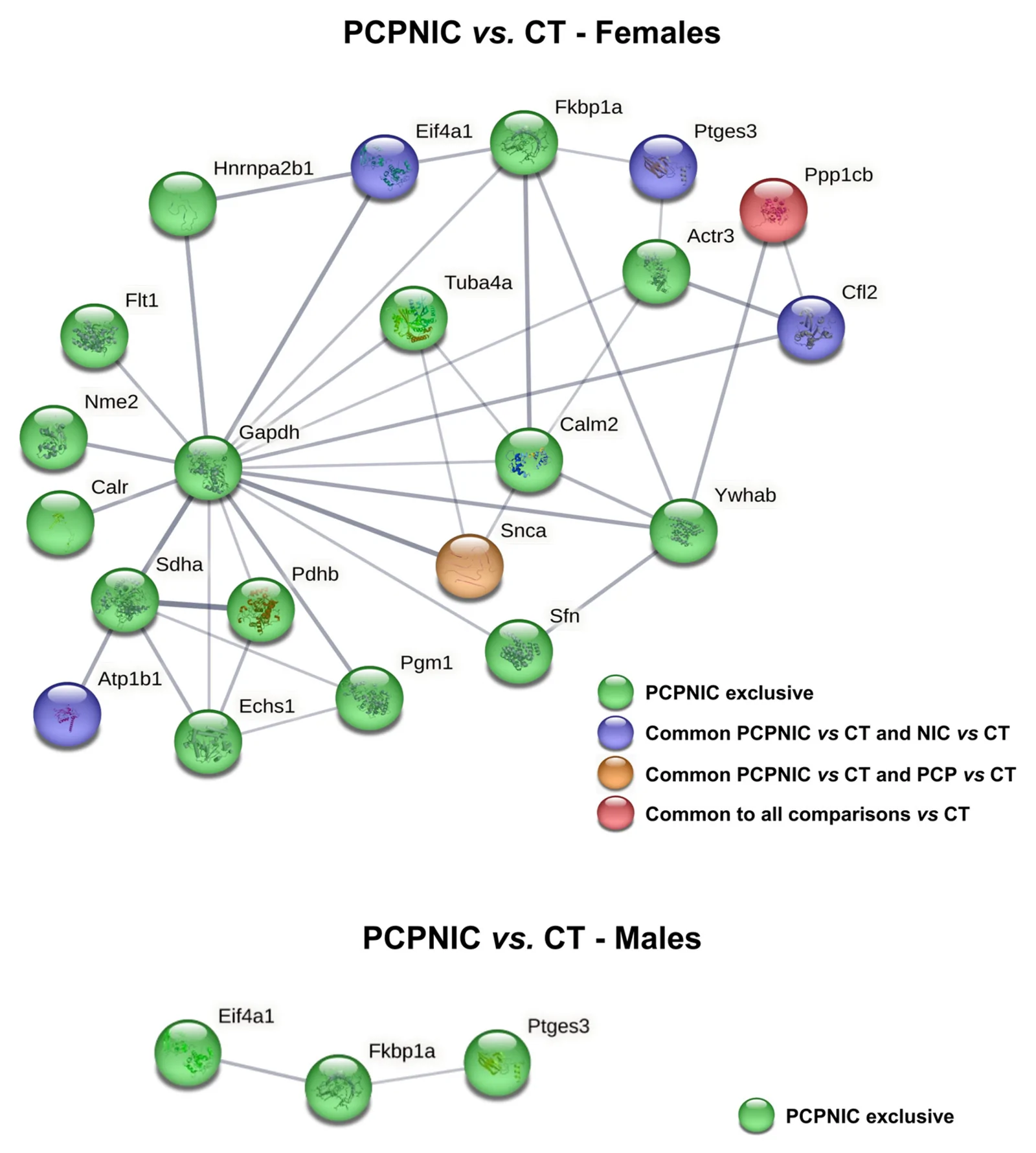
Interaction network of differentially abundant proteins in the dorsal striatum of female (**top image**) and male (**bottom image**) mouse models of the comorbidity (PCPNIC) when compared to controls (CT). Each node represents a protein identified by proteomic analysis, displayed using its corresponding mouse gene symbol, as required by STRING. Lines represent protein–protein associations, and line thickness indicates the strength of evidence supporting each association. The protein structures depicted within the nodes are generated by STRING. Exclusively differentially expressed proteins in mouse models of the comorbidity are shown in green. Purple indicates proteins commonly differentially expressed in both schizophrenia (PCP) and PCPNIC models, while orange identifies proteins commonly differentially expressed in nicotine use (NIC) and PCPNIC models. Proteins differentially expressed in all three conditions are shown in red. The proteins were analyzed by STRING, and the network was built using ‘medium confidence’ (0.4) interaction score.

To further understand the impact of combined nicotine and phencyclidine in the dorsal striatum of adolescent mice, PCPNIC differentially expressed proteins were accommodated in interactome maps. The female interactome map consisted of 21 proteins forming one interconnected network. These included 15 exclusively differentially expressed proteins in the PCPNIC group, four commonly differentially expressed proteins in the PCPNIC and NIC groups, one commonly differentially expressed protein in the PCPNIC and PCP groups and one commonly differentially expressed protein in all groups (Figure 6 upper panel). Glyceraldehyde-3-phosphate dehydrogenase (GAPDH) stood out as centrally located, being connected to 17 of the PCPNIC differentially abundant proteins. In males, the interactome map comprised three proteins exclusively differentially expressed in the PCPNIC group (Figure 6 lower panel). Peptidyl-prolyl cis-trans isomerase (FKBP1A) connected the other two proteins, Prostaglandin E synthase 3 (PTGES3) and Eukaryotic initiation factor 4A-I (EIF4A1). Notably, all three proteins were also present and interconnected in the female interactome map.

## 3. Discussion

The present study provides evidence of altered proteome profiles in the dorsal striatum of female and male adolescent mouse models of SCHZ with and without a history of nicotine exposure. In females, there was a higher number of proteins differentially expressed in PCPNIC mice (PCPNIC vs. CT) when compared to the other groups (PCP vs. CT and NIC vs. CT), indicating that the combined insults resulted in a greater disruption of the dorsal striatal proteome than either condition alone. In males, the comparison between differentially expressed proteins in PCP (PCP vs. CT) and PCPNIC (PCPNIC vs. CT) mice showed a higher number of differentially expressed proteins in the dorsal striatum of co-exposed mice, suggesting that a history of nicotine during adolescence exacerbates the proteomic alterations associated with the SCHZ model. However, despite the relatively short exposure period, nicotine alone led to more pronounced alterations in protein expression (NIC vs. CT) than those observed in both PCP and PCPNIC groups. This effect suggests that, in co-exposed males, the combined effects of phencyclidine and nicotine led to mitigation of the effects of nicotine history only.

These observations are consistent with previous reports describing sex-related differences in SCHZ patients [49,50,51,52], in animal models of SCHZ [11,12,47,53], as well as in smokers [54] and rodents exposed to nicotine [55,56,57,58]. Sex-specific patterns also emerged from the analysis of proteins that were commonly or exclusively differentially abundant, leading to distinct Reactome pathway enrichments and protein–protein interaction networks in females and males. However, despite the sex-dependent differences in individual protein alterations, there were biological targets shared between sexes in the comorbidity model. Notably, the analysis of exclusively differentially expressed proteins revealed enrichment in broad Reactome categories related to the immune system, metabolism and signal transduction in both female and male mice. These findings are consistent with previous reports in the prefrontal cortex and nucleus accumbens [11,12]. The overlap at the level of biological processes indicates that common functional domains may be disrupted in males and females despite distinct sex-specific molecular signatures.

### 3.1. Exclusively Differentially Abundant Proteins in Pcpnic Mice

Exclusively differentially abundant proteins in PCPNIC mice may indicate subthreshold effects of nicotine history and phencyclidine that, when combined, lead to disrupted expression of proteins. However, it is also possible that the combined insults engage biological processes that differ qualitatively from those elicited by either nicotine or phencyclidine alone, resulting in a distinct molecular profile. These possibilities are consistent with previous findings from our group [10,11,43,59].

In both females and males, Reactome pathway analysis revealed enrichment in immune system, signal transduction, and metabolism, consistent with previous findings. Immune dysfunction is widely recognized as a key component of SCHZ pathophysiology [60], and both tobacco smoke and, more recently, electronic cigarettes were shown to modulate immune function [61], at least in part through the effects of nicotine [62]. Similarly, metabolic dysregulation is a well-established feature of both SCHZ and tobacco use, and alterations in signal transduction pathways further intersect these processes [63,64,65,66,67]. Notably, these same pathways were previously reported as exclusively differentially abundant in the medial prefrontal cortex and nucleus accumbens of female and male mouse co-models of SCHZ and nicotine use [11,12], which supports the possibility of convergent biological mechanisms underlying the comorbidity.

It should be noted, however, that sex differences have been well documented in SCHZ, with women showing a greater prominence of certain positive symptoms and, more consistently, affective symptoms, whereas generally exhibiting a more favorable course, with less severe cognitive and negative symptoms than men [50,68]. Sex differences are already evident at early stages of psychosis [69], and retrospective and follow-up studies of patients with SCHZ further indicate fewer premorbid deficits and less pronounced functional decline in women than in men [50]. Sex differences have also been reported in responses to nicotine, with women experiencing greater difficulty maintaining abstinence, while some evidence suggests that men may exhibit greater sensitivity to the pharmacological reinforcing effects of nicotine [70,71]. Although the mechanisms underlying these sex-dependent differences are likely multifactorial, the identification of sex-specific pathways opens a range of possibilities for investigating biological processes relevant to SCHZ-related alterations and the neural adaptations induced by nicotine exposure in each sex.

In this context, female-specific pathways, including those related to signaling by Rho GTPases, vesicle-mediated transport, membrane trafficking, and transcriptional regulation by TP53, may be functionally interconnected and may contribute to processes involved in synaptic function and neuroplasticity. Rho-family GTPases coordinate synaptic development and plasticity by regulating actin and microtubule cytoskeletal dynamics, membrane trafficking, and gene expression, in part via Rho-kinase [72,73]. Notably, several components of Rho signaling are regulated by TP53, further supporting a coordinated network linking these processes [74]. Variants in genes involved in GTPase RhoA signaling have been associated with the development of SCHZ, and Rho-kinase inhibitors exhibit antipsychotic-like effects, ameliorating cognitive impairment and rescuing social interaction impairment and prepulse inhibition deficits in animal models of the disorder [73,75]. Although evidence linking nicotine to Rho signaling is more limited, interactions between α7 nicotinic acetylcholine receptors and G proteins have been shown to activate RhoA GTPase, resulting in decreased actin polymerization and neurite outgrowth [76].

Regarding male-specific enriched pathways, risk alleles related to apoptosis have been associated with SCHZ, and accumulating evidence highlights a central role of the mitochondrial apoptotic machinery in its etiopathogenesis, suggesting that dysregulation of apoptosis is a key factor in disease onset [77]. In the context of nicotine exposure, both anti- and pro-apoptotic effects have been described [78], with neurotoxic effects particularly evident during adolescence [79,80].

### 3.2. Commonly Differentially Abundant Proteins in Pcpnic Mice

The contribution of phencyclidine exposure to the profile of PCPNIC differentially abundant proteins in the dorsal striatum was limited, comprising one protein upregulated in females and one protein downregulated in males. Given that some Reactome enrichments were supported by only one or a few differentially expressed proteins, these analyses are best interpreted as descriptive and hypothesis generating, highlighting biological processes that merit further investigation rather than demonstrating definitive pathway-level alterations.

Four Reactome pathways were identified in the enrichment analysis of PCP and PCPNIC females, all related to the immune system. Even though Alpha-synuclein (SNCA), a nuclear and presynaptic neuronal protein, has been classically involved in the trafficking of synaptic vesicles and regulation of neurotransmitter exocitosis [81] and gene transcription involved in neuronal differentiation [82], there is also support for immune-related roles of this protein. Increased SNCA expression has been implicated in the recruitment of immune cells [82]. Moreover, SNCA may act as an activator of sterile immune responses, and in vitro, it has been shown to increase levels of inflammatory mediators in microglia [82]. There are few reports linking the SNCA protein to SCHZ. In a study examining SNCA single-nucleotide polymorphisms in patients with SCHZ from a northern Han Chinese population, the frequency of the ATT haplotype was higher in female patients when compared to controls [83]. We failed to find studies in high risk or first episode individuals. However, in patients with late-onset SCHZ, SNCA was increased in CD45+ white blood cells [84]. While these data suggest the involvement of SNCA in SCHZ pathophysiology, further studies are needed to investigate its roles in this disorder and in the comorbidity.

Regarding male mice, pathways involved with the release cycle of classic neurotransmitters were enriched in both PCP and PCPNIC. Consistent with this finding, the sole protein differentially abundant, Syntaxin-1A (STX1A), is a protein of the SNARE complex in presynaptic terminals that mediates synaptic vesicle fusion and neurotransmitter release [85]. Postmortem analysis of the ventromedial caudate in patients with SCHZ has shown decreased Syntaxin levels, accompanied by increased Syntaxin–SNAP-25 interactions [86]. This pattern, consistent with increased SNARE complex formation and impaired exocytosis and vesicle recycling [87], has been proposed as a compensatory mechanism aimed at attenuating the excessive dopamine release characteristic of SCHZ [86]. STX1A downregulation was recently shown to occur in the nucleus accumbens of adolescent mice modeled to the comorbidity [12]. However, this is the first study that identifies the same pattern in the dorsal striatum. Overall, these findings reinforce the neurotransmitter systems imbalance as central to SCHZ [88,89] and, importantly, also to the comorbidity.

The contribution of nicotine history to the dorsal striatum proteomic alterations observed in PCPNIC mice, represented by proteins commonly altered in both NIC and PCPNIC groups, comprised four proteins in females and two in males. As observed for the phencyclidine-associated contribution to the PCPNIC proteomic profile, the pathway enrichments, particularly in males, were driven by a small number of differentially abundant proteins. Therefore, these findings should be considered preliminary and interpreted with caution.

In females, Reactome pathways related to stress-induced heat shock responses as well as immune and inflammatory processes were enriched. These processes are often co-activated in response to environmental and endogenous compounds that induce proteotoxic stress [90]. Heat shock proteins were previously shown to be differentially expressed in the nucleus accumbens and amygdala of adult rats exposed to nicotine [91] and in the medial prefrontal cortex and nucleus accumbens of adolescent mice co-modeled to SCHZ and nicotine use [11,12]. The current findings are also consistent with evidence of the involvement of an altered inflammatory status and enhanced immune system signaling in the pathogenesis of nicotine misuse [92,93].

In males, the enrichment of pathways involved in mitochondria fatty acid beta oxidation and in branched-chain amino acid catabolism was driven by the deregulation of Enoyl-CoA hydratase mitochondrial (ECHS1), whereas the enrichment of translation initiation pathways was associated with the deregulation of Translation initiation factor Eif-2B subunit epsilon (EIF2B5). ECHS1 is a multifunctional enzyme that catalyzes fatty acid beta oxidation and the hydration of intermediates in branched-chain amino acid catabolism [94]. Accordingly, its downregulation in PCPNIC males suggests impaired energy production, consistent with evidence implicating disrupted energy metabolism in the pathophysiology of SCHZ [95]. Supporting this possibility, case reports provide evidence that a ketogenic diet, characterized by high fat content, may lead to remission of psychotic symptoms in SCHZ patients and, in animal models, to reverse sensorimotor gating deficits, improve working memory, and normalize social behaviors [95]. In turn, EIF2B is a complex essential for protein synthesis. EIF2B recycles EIF2-GDP to its active form EIF GTP, enabling further rounds of translation initiation [96]. Although we found no direct evidence linking SCHZ and nicotine exposure to EIF2B5, reduced activity of EIF2α, which is modulated by EIF2B5 [96], has been pointed out as responsible for an increased susceptibility of adolescent mice to changes in synaptic strength caused by nicotine [97].

Common contributions of PCP and NIC to the PCPNIC dorsal striatum profile of differentially abundant proteins were only identified in females. Three out of four differentially expressed proteins were enriched in pathways involved in the resolution of inflammation, cytoskeletal rearrangements, and cell survival and proliferation. Among them, Serine/threonine-protein phosphatase PP1-beta catalytic subunit (PPP1CB) encodes one of the three catalytic subunits of protein phosphatase 1 (PP1), a key regulator of neuroplastic processes such as long-term depression [98]. Inhibition of PP1 enhances dopaminergic signaling, a mechanism implicated in both SCHZ and addiction [99]. PPP1CB was also differentially expressed in the medial prefrontal cortex of a mouse co-model of SCHZ and nicotine use [11], suggesting that it could be of interest as a candidate marker of the comorbidity, associated with cytoskeletal remodeling and dopaminergic imbalance. Another differentially expressed protein, Glutathione S-transferase Mu 4 (GSTM4), is a member of the glutathione S-transferase superfamily. Its enrichment in pathways related to resolution of inflammation is consistent with the role of this enzyme family in maintaining central nervous system redox homeostasis through the detoxification of endogenous and exogenous compounds [100,101]. Postmortem studies have reported reduced GST Mu levels in the prefrontal cortex of SCHZ patients [102]. Accordingly, the downregulation of GSTM4 in the dorsal striatum of PCPNIC mice aligns with evidence implicating redox imbalance in the pathophysiology of SCHZ. Finally, ADP-ribosylation factor 5 (ARF5), a class II ARF family member [103], regulates vesicular trafficking and Golgi-to-endoplasmic reticulum transport, processes essential for cell homeostasis and survival [104]. To our knowledge, this is the first report of ARF5 deregulation in an animal model combining SCHZ-related and nicotine exposure-related insults.

### 3.3. Interactome Maps of Pcpnic Mice

STRING-based interactome maps are constructed from known and predicted protein–protein interactions; therefore, network connectivity should be interpreted as an indicator of potential functional relationships rather than direct evidence of causal or mechanistic relevance. Accordingly, proteins occupying central positions within these networks are discussed here as candidate nodes for further investigation.

The interactome map of adolescent females was organized into a single interconnected network comprising fifteen proteins exclusively differentially abundant in the PCPNIC group, four proteins shared between the PCPNIC and NIC groups, one shared between the PCPNIC and PCP groups, and one differentially abundant across all experimental groups. GAPDH occupied a central position, interacting with seventeen proteins.

GAPDH, a member of the glyceraldehyde-3-phosphate dehydrogenase protein family, catalyzes an important energy-yielding step in glycolysis, the oxidative phosphorylation of glyceraldehyde-3-phosphate [105,106]. Consistent with its role in cellular metabolism and energy production, two of GAPDH’s strongest interactome connections were with Phosphoglucomutase 1 (PGM1) and Succinate dehydrogenase [ubiquinone] flavoprotein subunit, mitochondrial (SDHA). PGM1 catalyzes the interconversion of glucose 1-phosphate and glucose 6-phosphate, linking glycolysis with glycogen and glycoconjugates [107]. SDHA, in turn, is the catalytic subunit of the mitochondrial complex II, functioning in both the citric acid cycle and electron transport chain. It converts succinate to fumarate and is essential for aerobic energy production in the mitochondria [108]. The deregulation of these interconnected proteins in the dorsal striatum interactome map is consistent with altered bioenergetic homeostasis in our comorbidity model and aligns with previous reports of disrupted energy metabolism in SCHZ [95] and nicotine exposure [109].

Beyond its canonical metabolic role, GAPDH is also recognized as a “moonlighting” protein that participates in a wide range of non-glycolytic cellular processes [105,106]. In this context, GAPDH’s robust connections with Heterogeneous nuclear ribonucleoproteins A2/B1 (HNRNPA2B1), SNCA, and EIF4A1 in the female map stood out. GAPDH is known to be an interaction partner with SNCA [110]. GAPDH–SNCA interaction leads to inactivation of GAPDH, glycolysis inhibition and initiation of apoptotic processes [111,112,113,114,115]. Accordingly, the link between these proteins shown in the female interactome map, while consistent with the established role of disrupted energy metabolism in SCHZ [95], also points to the involvement of apoptosis, increasingly recognized as a contributing factor to SCHZ progression [77,116]. In the context of nicotine exposure, pro-apoptotic effects have been described during adolescence [79,80]. Moreover, nicotine has been shown to modulate GAPDH expression [117] and to form a transient complex with SNCA, reducing its aggregation [118]. Collectively, these findings suggest that GAPDH–SNCA interactions converge on biological pathways implicated in both SCHZ- and nicotine-related insults, supporting shared mechanisms in the co-exposure model.

The connection between GAPDH and HNRNPA2B1 further points to potential links between metabolic regulation and RNA processing. HNRNPA2B1, a member of the heterogeneous nuclear ribonucleoprotein (HNRNP) A/B subfamily, is a multi-tasking regulator of gene transcription, RNA processing and post-transcriptional modification [119,120]. Notably, GAPDH and HNRNPA2B1 share several functional properties that may support cooperative physiological roles: both bind DNA and RNA, affecting mRNA stability and regulating protein expression at the translational level [121]. Altered expression of both proteins has been reported in cancer cells [122], and both bind to the 3′untranslated region of p53 mRNA [123]. While these findings raise the possibility of a functional interaction between GAPDH and HNRNPA2B1, future studies will be necessary to determine whether this interaction is relevant in the context of combined SCHZ- and nicotine-related insults.

Interestingly, the third protein strongly connected to GAPDH also showed a robust link to HNRNPA2B1. EIF4A1 is a component of the EIF4F translation initiation complex, responsible for the RNA helicase activity [124]. HNRNPA2B1 and EIF4A1 are critical RNA-binding proteins with complementary roles in mRNA regulation. HNRNPA2B1 participates in mRNA splicing, trafficking, modification and modulation of translation initiation [120]. EIF4A1, in turn, promotes translation initiation [125]. Functional connections between EIF4A1 and GAPDH remain poorly explored. Although both proteins have been shown to assemble into functional translational regulatory complexes in non-neuronal cells [126], this is the first study that reports GAPDH and EIF4A1 deregulation in association with combined SCHZ-related and nicotine exposure-related insults.

In adolescent males, the interactome map was composed of three proteins exclusively differentially abundant in the PCPNIC group. Peptidyl-prolyl cis-trans isomerase (FKBP1A), Prostaglandin E synthase 3 (PTGES3) and EIF4A1. Although functionally distinct, FKBP1A, PTGES3, and EIF4A1 converge on proteostasis-related processes, contributing to translation initiation, protein folding, chaperone-mediated stabilization, and intracellular protein trafficking. As mentioned previously, Eif4a1 provides the RNA helicase activity required for translation initiation [124]. PTGES3, in turn, functions as an HSP90 co-chaperone, inhibiting HSP90 ATPase activity and stabilizing the client-bound conformation of the complex [127]. FKBP1A connected the other two proteins; however, this apparent centrality should be interpreted cautiously, as it is dependent on the limited number of proteins included in the male network. FKBP1A belongs to the FK506-binding protein (FKBP) family, a subgroup of immunophilins within the broader Peptidyl-prolyl isomerase (PPIase) chaperone superfamily. These enzymes catalyze proline cis–trans isomerization, thereby influencing protein folding, stability, activation, and degradation [128]. Beyond their chaperone activity, FKBP proteins participate in multiple cellular processes, including gene expression regulation, intracellular protein transport, and signaling pathways [129]. In the male map, FKBP1A was downregulated relative to the CT group. Previous studies have shown that selective depletion of FKBP1A in the forebrain enhances obsessive–compulsive-like perseverative behaviors and memory, as well as protein synthesis-dependent long-term potentiation [130]. FKBP1A was found differentially expressed in the prefrontal cortex of adolescent female mice co-modeled to SCHZ and nicotine use [11]. However, to our knowledge, this is the first report of deregulation in the dorsal striatum and in males. The functional implications of these alterations remain to be elucidated. Notably, all three proteins were also present and interconnected in the female map, suggesting that disruption of protein homeostasis mechanisms may represent a common feature underlying the pathophysiology of SCHZ and nicotine comorbidity.

### 3.4. Limitations

Some limitations of the present study should be considered when interpreting the findings. First, nicotine and phencyclidine exposure differed in timing relative to tissue collection. Nicotine exposure ended several days earlier, whereas the final phencyclidine administration occurred on the day of euthanasia and brain collection. Therefore, the present proteomic profile may reflect relatively persistent effects of nicotine exposure, whereas phencyclidine-associated alterations may include both cumulative effects of the exposure regimen and acute molecular responses to the final administration. Second, even though nicotine is considered the most important psychoactive substance present in tobacco and the one responsible for nicotine misuse disorder [131], tobacco smoke and ENDS contain numerous additional constituents. Accordingly, the alterations identified in the NIC and PCPNIC groups may not fully reflect the molecular effects of tobacco smoke or ENDS vapor exposure. Third, the proteomic analyses were performed using pooled dorsal striatum samples. Pooling allowed sufficient protein yield for mass spectrometry analysis; however, it may have reduced the assessment of biological variability across animals. Fourth, a relatively small number of biological replicates was used for proteomic analyses. Although rigorous filtering criteria were applied, including fold-change thresholds, coefficient of variation filtering, and FDR control for protein identification and for differential abundance testing, the possibility of false-positive or false-negative findings cannot be excluded. Fifth, the relatively modest proteome coverage obtained in this study may have limited the detection of additional differentially expressed proteins. This coverage reflects, in part, the use of the reviewed UniProt *Mus musculus* reference proteome databank, which was intentionally selected to prioritize high-confidence protein identification through curated entries rather than maximize the total number of identified proteins. As a result, Reactome pathway enrichment analyses were in some cases driven by a limited number of differentially expressed proteins, warranting cautious interpretation of pathway-level conclusions. These analyses are intended to provide biological context until confirmed by independent studies. Finally, the current data have not been validated using complementary experimental approaches. Therefore, the identified proteins and pathways should be regarded as hypothesis-generating and interpreted with caution. Future studies employing molecular techniques are needed to confirm the obtained results and to further elucidate the mechanisms associated with PCPNIC combined insults.

## 4. Materials and Methods

### 4.1. Materials

Ketamine (CEVA, Brazil), xylazine (Syntec, Brazil), flunixin (UCB/VET, Brazil), and enrofloxacin (VENCO, Brazil) were specific for veterinary use. Nicotine free base, dithiothreitol (DTT), iodoacetamide (IAA), formic acid, sodium deoxycholate and triethylammonium bicarbonate buffer (TEAB) were purchased from Sigma Chemical Co. (St. Louis, MO, USA). Phencyclidine was purchased from Alomone Labs (Jerusalem, Israel). Osmotic minipumps came from Alzet (Cupertino, CA, USA). Promega Biotecnologia do Brasil (São Paulo, Brazil) was the source of trypsin, and Thermo Scientific (Waltham, MA, USA) was the source of the resin column POROS 20 R2 and Qubit protein assay kit. The nanoEase Symmetry C18 (5 µm, 180 µm × 20 mm) TRAP column, the nanoAcquity BEH130 C18 (1.7 µm, 75 µm × 100 mm) reversed-phase column, [Glu1]-Fibrinopeptide B human (GFP) and Total Recovery vials were purchased from Waters Corporation, Milford, MA, USA. Acetonitrile came from Merck HGaA (Darmstadt, Germany).

### 4.2. Animals and Treatment

The experimental procedures were approved by the Institute of Biology/UERJ Ethical Committee for Animal Research (protocol#: CEUA/033/2018), minimizing the number of animals used and avoiding animal suffering, in accordance with Brazilian Law # 11.794/2008. All mice were kept in our animal facility housed in groups of 2–5 at 21–22 °C on a 12 h light/dark cycle (lights on at 1:00 a.m.). Access to food and filtered water was *ad libitum*. Animals were derived from a C57BL/6 colony maintained at the *Universidade Federal Fluminense* (Niteroi, Brazil) for over 65 generations.

Additional details on the experimental protocol are available elsewhere [9]. Mice were exposed to a nicotine solution or milli-Q water from postnatal day (PN) 37 to PN44. On PN36, osmotic minipumps (model 1007d; ALZET LLC, Campbell, CA, USA) were filled either with milli-Q water or nicotine-free base diluted in milli-Q water (pH 6.0) to deliver an initial dose rate of 24 mg/kg of nicotine per day. At PN37, mice were anesthetized with xylazine (20 mg/kg, i.p.) and ketamine (100 mg/kg, i.p.), a small area on the back was shaved, an incision was made to permit s.c. insertion of the minipump, and the incisions were sutured. For pain management and infection prevention, mice were given s.c. injections of Flunixin (2.5 mg/kg) and Enrofloxacin (2.5 mg/kg) right after closure and on the following day. Animals were allowed to recover from surgery in their home cages. The period of exposure to nicotine was intended to parallel human exposure during adolescence, a key period for initiation of tobacco and electronic nicotine delivery systems (ENDS) consumption [5,132,133]. The dose rate used in the current study results in nicotine serum levels [45] that are within the range of those found in smokers [134,135].

From PN38 to PN53, the animals were daily exposed to phencyclidine (PN38-52: 2.5 mg/kg; PN53: 10 mg/kg s.c.), an NMDA receptor antagonist used to model SCHZ [136,137,138]. The period of phencyclidine exposure was chosen based on evidence that the prodromal stage of the disorder begins early, still during adolescence [139,140,141], and that the first psychotic event in SCHZ frequently occurs between late adolescence and early adulthood [141,142]. The 10 mg/kg challenge on PN53 followed the experimental protocol established in our previous studies [11,12], allowing direct comparison with our earlier proteomic findings obtained using the same animal model. Control mice received a saline solution (NaCl 0.9%, s.c.). Accordingly, there were four experimental groups: CT (control), PCP (phencyclidine-exposed), NIC (nicotine-exposed), and PCPNIC (phencyclidine- and nicotine-exposed).

At PN53, after the end of nicotine exposure but still during the period of exposure to phencyclidine, mice were decapitated, and the brains were fast frozen in liquid nitrogen and stored at −76 °C. The 9-day interval between the end of nicotine exposure and euthanasia aimed to assess lasting effects of nicotine without the presence of confounding factors associated with acute withdrawal signs [55,143,144]. Because euthanasia was performed on the same day as the last phencyclidine injection, the proteomic profile of mice exposed to phencyclidine may reflect both persistent alterations induced by the subchronic treatment and acute molecular effects of the last dose. Samples were punched from the frozen brains using a cryostat (Hyrax C25, Zeiss, Oberkochen, Germany) and a cylindric brain puncher (internal diameter 1.5 mm). The length and location of the punches followed the coordinates described in the Paxinos and Franklin stereotaxic atlas [145]: 0.48 mm of length (starting at 1.34 mm anterior to Bregma), aiming the dorsal striatum. The samples were frozen at −76 °C for posterior proteomics analysis.

### 4.3. Protein Extraction

To guarantee minimum sample concentration for the analysis, pools of samples from different mice were used instead of individual samples. Punches of the dorsal striatum from two animals within each experimental condition and sex were pooled to generate one biological sample. Thus, eight animals from each experimental group and sex were used to generate four independent biological replicates (four pools), with each pool representing two animals. Each biological sample was analyzed in three technical replicates, which were averaged before statistical analysis. Each sample pool was extracted with 500 μL of buffer (50 mM TEAB pH 8.5, 0.1% sodium deoxycholate). The samples were briefly vortexed and then incubated in an ultrasonic bath (SolidSteel, Piracicaba, SP, Brazil) for 30 min at 6 °C. After centrifugation at 18,400× *g* for 20 min at 4 °C (Eppendorf, centrifuge 5427R, Hamburg, Germany), the supernatants were transferred to new identified tubes. The extraction was performed twice, and the respective supernatants were merged. Acetonitrile with 0.1% formic acid was added to the supernatant for protein precipitation in a 4:1 proportion (*v*/*v*), centrifuged at 18,400× *g* for 10 min and 4 °C and washed twice with cold acetone. Proteins were resuspended in TEAB 50 mM pH 8.5, quantified using a Qubit^®^ fluorometer (Invitrogen, Carlsbad, CA, USA) and processed as described previously [11,12].

### 4.4. Protein Digestion and Nanoulpc-Mse Analysis

Forty micrograms of proteins was treated with 0.01% of sodium deoxycholate, reduced by the addition of 10 mM DTT at 56 °C for 30 min and alkylated with IAA 20 mM at 25 °C for 30 min. Afterward, samples were digested with sequencing grade trypsin in 1:100 trypsin:protein ratio in TEAB 50 mM pH 8.5 for 18 h at 37 °C under agitation on a Thermomixer Comfort (Eppendorf, Hamburg, Germany). Peptides were desalinated and concentrated through reverse phase chromatography with a resin column POROS 20 R2 and eluted with 7:3 acetonitrile:water with 0.05% formic acid. The peptides were dried, resuspended in 0.1% formic acid, and quantified (Qubit^®^ protein assay kit, Invitrogen), and the supernatants were transferred to Waters Total Recovery vials (Waters Corporation).

The nanoUPLC analysis of tryptic peptides was carried out using a nanoACQUITY UPLC system (Waters Corp., Milford, MA, USA) coupled to a Synapt G2-Si high-definition mass spectrometer (Waters Corp., Manchester, UK). The chromatographic system was equipped with a nanoEase Symmetry C18 (5 µm, 180 µm × 20 mm) TRAP column and a nanoAcquity BEH130 C18 (1.7 µm, 75 µm × 100 mm) reversed-phase column. Mobile phase A consisted of water, and mobile phase B consisted of acetonitrile, both with 0.1% (*v*/*v*) formic acid. The peptides were separated using mass spectrometry experimentation (MSE) chromatographic methods (gradient of 3 to 50% of B for 60 min; followed by a cleaning column gradient of 50 to 85% of B for 1 min; maintained in 85% of B for 5 min; then from 85 to 3% of B for 1 min). The flow rate was 450 ηL.min^−1^. The analytical column temperature was maintained at 35 °C, and the sample manager temperature was 9 °C. The lock mass was derived from the auxiliary pump using a constant flow rate of 500 ηL.min^−1^ at a concentration of 200 fmol of GFP to the reference sprayer of the NanoLockSpray source (Waters Corporation).

The mass spectrometer was operated in the resolution mode for all measurements. All analyses were carried out using nano-electrospray ionization in the positive ion mode (nanoESI+) and a NanoLockSpray ionization source. The lock mass channel was sampled at a frequency of 30 s. The ion source block temperature was set to 100 °C, and capillary voltage was set to 3 kV. The time-of-flight analyzer of the mass spectrometer was calibrated with an MS/MS spectrum of GFP solution. The final instrument calibration was obtained using the signal of GFP double charged precursor ion [M + 2H]2+ = 785.8426. The exact mass retention time from multiplexed data-independent acquisition (DIA) scanning MSE analysis was collected in an alternating low energy and elevated energy acquisition mode. In the elevated collision energy mode, the collision energy was increased from 15 to 55 eV applied to the trap “T-wave” collision-induced dissociation cell with argon gas. The radio frequency was adjusted such that the ions were effectively acquired from *m*/*z* 50 to 2000, which ensured that any masses less than 50 *m*/*z* observed in the elevated-energy spectra were only derived from dissociations in the collision cell.

### 4.5. Data Processing

#### 4.5.1. Protein Identification

To identify and quantify peptides and proteins, the MS data were processed and searched using the Progenesis QI for Proteomics version 4.2 (Nonlinear Dynamics, Newcastle upon Tyne, UK). The reviewed UNIPROT protein databank release 2022-03 (http://www.uniprot.org) with specific annotations for *Mus musculus* was used for database searching (~17,000 curated protein entries). A reverse database was generated for monitoring the false-positive rate (false discovery rate—FDR). The parameters for database searching were tryptic peptides with a maximum of two missed cleavages, maximum protein mass of 650 kDa, carbamidomethylation of cysteine as fixed modification, and oxidation of methionine as variable modification. The peptide mass error tolerance was set at 10 ppm, and the fragment mass error tolerance was set at 20 ppm. Peptide identification was accepted at FDR < 1%. Regarding protein identification, it required at least two peptides per protein, with a minimum of two fragment ions per peptide and three fragment ions per protein. In addition, protein identifications were required to have a mass accuracy < 20 ppm and a score > 10. Thus, proteins identified based on a single peptide were not included in the dataset.

#### 4.5.2. Normalization and Relative Quantification

Each biological sample was analyzed in three technical replicates. Protein abundance data were normalized in Progenesis QI using the “Normalize to all proteins” procedure, which operates on ratiometric data in log space; no additional log transformation was applied before statistical analysis. Relative protein quantification was then performed using the Hi3 (Top3) approach, in which protein abundance is estimated using the three most intense quantifiable peptides assigned to each protein, as described previously [146]. To ensure the robustness of protein quantification, proteins represented by fewer than three quantifiable peptides, although retained in the list of identified proteins, were excluded from the quantitative analysis. Briefly, following the identification step, the abundance of each peptide was calculated from all constituent peptide ions. The ranking of peptide abundance was based on the integrated peptide abundance across all experimental runs, enabled by the absence of missing values in the three technical replicates and accurate alignment of the peptide signals. This approach allowed peptide selection to take all runs into account, providing a strong and consistent basis for protein abundance estimation. Finally, for each protein, three of the most abundant peptides (n = 3) were selected, and their abundances were averaged to provide the protein-level abundance estimate.

#### 4.5.3. Data Filtering

Only proteins detected in at least three of the four biological replicates within a given experimental condition and sex were retained for subsequent analyses. Missing values were not imputed; instead, Progenesis QI uses retention-time alignment and an ion map generated from aligned LC–MS runs to facilitate peptide ion detection and quantification across samples, including those with low signal intensity. The coefficient of variation (CV) was calculated across biological replicates, after averaging technical replicates, and proteins with CV ≤ 30% within each group were retained. Only proteins fulfilling all identification, quantification, replicate-detection, and CV criteria were included in the final analyses.

#### 4.5.4. Differential Abundance Analysis

Pairwise comparisons of each exposed group (PCP, NIC, and PCPNIC) with the CT group were performed using one-way analysis of variance (ANOVAs), aiming to identify proteins differentially abundant in each experimental condition. For the analyses, mice were a priori separated by sex, and the effects observed in females and males were not statistically compared. This approach allowed the characterization of proteomic alterations observed within each sex, as well as those shared between sexes, associated with each experimental condition. Multiple testing correction was applied using the Progenesis QI FDR procedure, separately for each pairwise comparison. Proteins were considered differentially abundant when they showed a fold change > 1.5 (increased or decreased) and an FDR-adjusted *p*-value (q) < 0.05.

#### 4.5.5. Downstream Analyses

Reactome pathways analysis was performed using the online Protein Analysis Through Evolutionary Relationships (PANTHER) classification system (http://www.pantherdb.org) [147]. The jvenn tool online (http://jvenn.toulouse.inra.fr/app/index.html; accessed on 12 May 2026) was used to draw Venn diagrams to visualize intersections among proteome datasets (https://bioinfogp.cnb.csic.es/tools/venny/index.html, accessed on 12 May 2026). Protein–protein interactome maps were generated using STRING (https://string-db.org/, http://jvenn.toulouse.inra.fr/app/index.html; accessed on 28 May 2026) [148] using the full STRING network, a medium minimum interaction confidence score (0.4), and all available evidence channels. Disconnected nodes were hidden from the displayed networks.

#### 4.5.6. Shared and Exclusive Proteins Across Exposure Groups

Potential contributions of phencyclidine exposure and nicotine history to the profile of PCPNIC differentially abundant proteins were explored by identifying proteins altered in both PCP and PCPNIC groups, as well as in NIC and PCPNIC ones, respectively. Differentially expressed proteins in all three drug-exposed groups indicate common contributions of phencyclidine and nicotine to the PCPNIC profile. The exclusive proteins, defined as the ones differentially abundant only in a given experimental group, were also identified: proteins exclusively differentially abundant in the PCPNIC group represent molecular alterations associated with the combined exposure to nicotine and phencyclidine that were not detected in either exposure alone. In turn, proteins exclusively differentially expressed in the NIC and PCP groups point to alterations associated with nicotine history or phencyclidine, respectively, that are not relevant to the outcomes of the combined insult; therefore, all related data are provided in Supplementary Material (Supplementary Material File S1—Sheets S12–S15).

## 5. Conclusions

The present study provides preliminary evidence that adolescent co-exposure to phencyclidine and nicotine induces proteomic alterations in the dorsal striatum, a region not classically emphasized in SCHZ and nicotine misuse research but increasingly recognized as relevant to habit formation, cognitive flexibility, and dopaminergic dysfunction [21]. Our exploratory findings suggest that the molecular profile of this comorbidity model differs between sexes, with females showing greater proteomic disruption under combined exposure and males displaying a distinct pattern in which adolescent nicotine exposure and phencyclidine produced a unique signature, characterized by amplification of SCHZ-associated alterations alongside partial mitigation of nicotine-driven molecular effects.

Despite sex-specific differences, convergent biological features emerged across both sexes, particularly involving pathways related to immune signaling, metabolic regulation, and signal transduction. The identification of proteins associated with synaptic transmission, bioenergetic regulation, RNA processing and apoptotic signaling suggests that dorsal striatal dysfunction in this comorbidity model extends beyond classical neurotransmitter imbalance and involves broader disruptions of cellular adaptive mechanisms, which corroborates previous findings in the prefrontal cortex and nucleus accumbens, brain regions classically involved in the pathophysiology of SCHZ and nicotine misuse [11,12]. Notably, these data might be associated with previous behavioral findings. Using this same combined insult to model SCHZ–nicotine comorbidity, nicotine history during adolescence was shown to mitigate positive-like and negative-like behaviors elicited by phencyclidine, while memory and sensorimotor gating deficits evoked by phencyclidine were neither improved nor worsened by nicotine history [9].

Finally, the interactome analyses highlighted GAPDH in females and an FKBP1A-centered network in males as candidate network-associated proteins. Because network connectivity does not demonstrate a functional or causal role, these proteins should be viewed as hypotheses for further investigation of how SCHZ-related and nicotine-related insults may converge in the dorsal striatum. Collectively, these results expand the current understanding of the neurobiological substrates potentially associated with SCHZ–nicotine comorbidity and position the dorsal striatum as a previously underappreciated target for mechanistic investigation. Integration of the molecular candidates identified here with complementary approaches within multimodal precision psychiatry frameworks [149] may help clarify their translational relevance. Future studies should determine the functional consequences of the identified proteomic alterations for this clinically relevant comorbidity.

## Data Availability

The mass spectrometry proteomics data were deposited in the ProteomeXchange Consortium via the PRoteomics Identification Database (PRIDE) partner repository (http://www.ebi.ac.uk/pride, accessed on 9 June 2026) with the dataset identifier PXD079429.

## Supplementary Materials

The following supporting information can be downloaded at: https://www.mdpi.com/article/10.3390/ijms27209328/s1.

## Abbreviations

The following abbreviations are used in this manuscript:

ARF5	ADP-ribosylation factor 5
CT	Control group
ECHS1	Enoyl-CoA hydratase, mitochondrial
EIF2B5	Translation initiation factor eIF-2B subunit epsilon
EIF4A	Eukaryotic initiation factor 4A-I
ENDS	Electronic nicotine delivery systems
FDR	False discovery rate
FKBP1A	Peptidyl-prolyl cis-trans isomerase
GAPDH	Glyceraldehyde-3-phosphate dehydrogenase
GSTM4	Glutathione S-transferase Mu 4
HNRNPs	Heterogeneous nuclear ribonucleoproteins
HNRNPA2B1	Heterogeneous nuclear ribonucleoproteins A2/B1
NIC	Nicotine-exposed group
NMDA	N-methyl-D-aspartate
PANTHER	Protein Analysis Through Evolutionary Relationships
PCP	Phencyclidine-exposed group
PCPNIC	Phencyclidine- and nicotine-exposed group
PGM1	Phosphoglucomutase 1
PN	Postnatal day
PTGES3	Prostaglandin E synthase 3
PPP1CB	Serine/threonine-protein phosphatase PP1-beta catalytic subunit
SCHZ	Schizophrenia
SDHA	Succinate dehydrogenase [ubiquinone] flavoprotein subunit, mitochondrial
SNCA	Alpha-synuclein
STX1A	Syntaxin-1A
TP53	Cellular tumor antigen p53
