# Supplementary material for: Beyond Classical Reward Circuits: Dorsal Striatum Proteomic Alterations in an Adolescent Phencyclidine-Induced Mouse Model of Schizophrenia and Nicotine Use
Source: Int J Mol Sci. 2026 Oct 20;27(20):9328. doi: 10.3390/ijms27209328 (PMC13705055; doi:10.3390/ijms27209328)
Supplement: Supplementary file 1 [file ijms-27-09328-s001.zip › ijms-4598366 Supplementary Material S2 - Figures R1.pdf]

SUPPLEMENTARY MATERIAL 2

International Journal of Molecular Sciences

Beyond Classical Reward Circuits: Dorsal Striatum Proteomic Alterations in an Adolescent Phencyclidine-induced Mouse Model of Schizophrenia and Nicotine Use

Thainá Pereira de Souza[1], Andrés Rodríguez-Vega[1], Ana Carolina Dutra-Tavares[2], Keila A. Semeão[1], Claudio Carneiro Filgueiras[1], Anderson Ribeiro-Carvalho[3], Alex Christian Manhães[1], Yael Abreu-Villaça[1*]

1. Laboratório de Neurofisiologia, Departamento de Ciências Fisiológicas, Instituto de Biologia Roberto Alcantara Gomes, Universidade do Estado do Rio de Janeiro (UERJ), Av. Prof. Manuel de Abreu 444, 5 andar – Vila Isabel, Rio de Janeiro, RJ, 20550-170, Brazil.

2. Departamento de Ciências Biomédicas e Saúde, Instituto de Biologia Roberto Alcantara Gomes, Universidade do Estado do Rio de Janeiro (UERJ), Cabo Frio 28905-320, RJ, Brazil.

3. Departamento de Ciências, Faculdade de Formação de Professores da Universidade do Estado do Rio de Janeiro, São Gonçalo, RJ, 24435-005, Brazil.

<u>Corresponding author:</u> Dr. Yael Abreu-Villaça
E–mail: yael_a_v@yahoo.com.br
yael.villaca@uerj.br
ORCID: 0000-0002-9801-6179

Short title: Adolescent nicotine and schizophrenia dorsal striatum proteomic profile

Supplementary figures S1 to S4. Data from differentially abundant proteins in the dorsal striatum of female and male mice. **Fig S1**: Exclusively differentially abundant in female (S1a-S1c) and male (S1d-S1e) mice modeled to the comorbidity (PCPNIC) when compared to controls (CT). **Fig S2**: Commonly differentially abundant in female (S2a) and male (S2b) mice modeled schizophrenia (PCP) and the comorbidity (PCPNIC), when compared to CT. **Fig S3**: Commonly differentially abundant in female (S3a) and male (S3b) mice modeled nicotine use (NIC) and the comorbidity (PCPNIC), when compared to CT . **Fig S4.** Commonly differentially abundant in all experimental groups when compared to CT. The panels show complete lists of Reactome pathways. Pathways in bold and underlined were shared between sexes. ▼ indicates that a given protein is downregulated in a given comparison, while ▲ indicates that a given protein is upregulated.

Exclusively differentially abundant in PCPNIC *vs.* CT

Actr3: Actin-related protein 3; **Cabp5:** Calcium-binding protein 5; **Calm2:** Calmodulin-2; **Calr:** Calreticulin; **Ceacam5:** Carcinoembryonic antigen-related cell adhesion molecule 5; **Csmd1:** CUB and sushi domain-containing protein 1; **Echs1:** Enoyl-CoA hydratase_mitochondrial; **Fkbp1a:** Peptidyl-prolyl cis-trans isomerase FKBP1A; **Flt1:** Vascular endothelial growth factor receptor 1; **Gapdh:** Glyceraldehyde-3-phosphate dehydrogenase; **Hnrnpa2b1:** Heterogeneous nuclear ribonucleoproteins A2/B1; **Nelfe:** Negative elongation factor E; **Nme2:** Nucleoside diphosphate kinase B; **Pdhb:** Pyruvate dehydrogenase E1 component subunit beta_mitochondrial; **Pgm1:** Phosphoglucomutase-1; **Rab6b:** Ras-related protein Rab-6B; **Sdha:** Succinate dehydrogenase [ubiquinone] flavoprotein subunit_mitochondrial; **Sfn:** 14-3-3 protein sigma; **Tmem191c:** Transmembrane protein 191C; **Tuba4a:** Tubulin alpha-4A chain; **Vnn3:** Vascular non-inflammatory molecule 3; **Ywhab:** 14-3-3 protein beta/alpha.

FEMALES

REACTOME PATHWAYS	# OF PTNS	Actr3	Calr	Echs1	Fkbp1a	Flt1	Gapdh	Nelfe	Nme2	Pdhb	Pgm1	Rab6b	Sdha	Sfn	Tuba4a	Ywhab
**REGULATION: PCPNIC*vs.* CT**		▲	▲	▼	▲	▼	▲	▲	▼	▲	▼	▼	▲	▲	▲	▲
**Immune system (R-MMU-168256)**	**7**	■	■		■				■		■				■	■
**Signal transduction (R-MMU-162582)**	**7**	■			■	■				■				■	■	■
**Metabolism (R-MMU-1430728)**	**6**			■			■		■	■	■		■			
RHO GTPase effectors (R-MMU-195258)	4	■												■	■	■
Signaling by Rho GTPases (R-MMU-194315)	4	■												■	■	■
Signaling by Rho GTPases, Miro GTPases and RHOBTB3 (R-MMU-9716542)	4	■												■	■	■
Vesicle-mediated transport (R-MMU-5653656)	4	■	■									■			■	
**Adaptive immune system (R-MMU-1280218)**	**4**		■		■										■	■
Transcriptional regulation by TP53 (R-MMU-3700989)	3							■						■		■
Membrane trafficking (R-MMU-199991)	3	■										■			■	
Cell cycle (R-MMU-1640170)	3													■	■	■
Generic transcription pathway (R-MMU-212436)	3							■						■		■
Regulation of localization of FOXO transcription factors (R-MMU-9614399)	2													■		■
Activation of BAD and translocation to mitochondria (R-MMU-111447)	2													■		■
Chk1/Chk2(Cds1) mediated inactivation of Cyclin B:Cdk1 complex (R-MMU-75035)	2													■		■
Activation of BH3-only proteins (R-MMU-114452)	2													■		■
RHO GTPases activate PKNs (R-MMU-5625740)	2													■		■
FOXO-mediated transcription (R-MMU-9614085)	2													■		■
**mTORC1-mediated signalling (R-MMU-166208)**	**2**				■											■
**Intrinsic pathway for apoptosis (R-MMU-109606)**	**2**													■		■
**MTOR signalling (R-MMU-165159)**	**2**				■											■
COPI-independent Golgi-to-ER retrograde traffic (R-MMU-6811436)	2											■			■	
Pyruvate metabolism and Citric Acid (TCA) cycle (R-MMU-71406)	2									■			■			
G2/M DNA damage checkpoint (R-MMU-69473)	2													■		■
TP53 regulates metabolic genes (R-MMU-5628897)	2													■		■
**Apoptosis (R-MMU-109581)**	**2**													■		■
Golgi-to-ER retrograde transport (R-MMU-8856688)	2											■			■	
**Programmed cell death (R-MMU-5357801)**	**2**													■		■
G2/M checkpoints (R-MMU-69481)	2													■		■
The citric acid (TCA) cycle and respiratory electron transport (R-MMU-1428517)	2									■			■			

Continues on the next page.

Exclusively differentially abundant in PCPNIC *vs.* CT

FEMALES (continuation)

REACTOME PATHWAYS	# OF PTNS	Actr3	Calr	Echs1	Fkbp1a	Flt1	Gapdh	Nelfe	Nme2	Pdhb	Pgm1	Rab6b	Sdha	Sfn	Tuba4a	Ywhab
**REGULATION: PCPNIC*vs.* CT**		▲	▲	▼	▲	▼	▲	▲	▼	▲	▼	▼	▲	▲	▲	▲
Intra-Golgi and retrograde Golgi-to-ER traffic (R-MMU-6811442)	2											■			■	
Metabolism of amino acids and derivatives (R-MMU-71291)	2			■						■						
Asparagine N-linked glycosylation (R-MMU-446203)	2		■												■	
Cell cycle checkpoints (R-MMU-69620)	2													■		■
Metabolism of carbohydrates (R-MMU-71387)	2						■				■					
Axon guidance (R-MMU-422475)	2	■													■	
Nervous system development (R-MMU-9675108)	2	■													■	
Beta oxidation of butanoyl-CoA to acetyl-CoA (R-MMU-77352)	1			■												
Galactose catabolism (R-MMU-70370)	1										■					
Neurophilin interactions with VEGF and VEGFR (R-MMU-194306)	1					■										
Beta oxidation of hexanoyl-CoA to butanoyl-CoA (R-MMU-77350)	1			■												
Beta oxidation of octanoyl-CoA to hexanoyl-CoA (R-MMU-77348)	1			■												
Beta oxidation of lauroyl-CoA to decanoyl-CoA-CoA (R-MMU-77310)	1			■												
Glycogen synthesis (R-MMU-3322077)	1										■					
Beta oxidation of decanoyl-CoA to octanoyl-CoA-CoA (R-MMU-77346)	1			■												
TP53 regulates transcription of genes involved in G2 cell cycle arrest (R-MMU-6804114)	1													■		
Calnexin/calreticulin cycle (R-MMU-901042)	1		■													
**Calcineurin activates NFAT (R-MMU-2025928)**	**1**				■											
VEGF ligand-receptor interactions (R-MMU-194313)	1					■										
VEGF binds to VEGFR leading to receptor dimerization (R-MMU-195399)	1					■										
Mitochondrial fatty acid beta-oxidation of saturated fatty acids (R-MMU-77286)	1			■												
Scavenging by class A receptors (R-MMU-3000480)	1		■													
Frs2-mediated activation (R-MMU-170968)	1															■
Glycogen breakdown (glycogenolysis) (R-MMU-70221)	1										■					
Ribavirin ADME (R-MMU-9755088)	1								■							
Prolonged ERK activation events (R-MMU-169893)	1															■
Rap1 signalling (R-MMU-392517)	1															■
N-glycan trimming in the ER and Calnexin/Calreticulin cycle (R-MMU-532668)	1		■													
Microtubule-dependent trafficking of connexons from Golgi to the plasma membrane (R-MMU-190840)	1														■	
Regulation of pyruvate dehydrogenase (PDH) complex (R-MMU-204174)	1									■						
Glycogen metabolism (R-MMU-8982491)	1										■					
Transport of connexons to the plasma membrane (R-MMU-190872)	1														■	
Butyrate Response Factor 1 (BRF1) binds and destabilizes mRNA (R-MMU-450385)	1															■

Continues on the next page.

Exclusively differentially abundant in PCPNIC *vs.* CT

FEMALES (continuation)

REACTOME PATHWAYS	# OF PTNS	Actr3	Calr	Echs1	Fkbp1a	Flt1	Gapdh	Nelfe	Nme2	Pdhb	Pgm1	Rab6b	Sdha	Sfn	Tuba4a	Ywhab
**REGULATION: PCPNIC *vs*. CT**		▲	▲	▼	▲	▼	▲	▲	▼	▲	▼	▼	▲	▲	▲	▲
Tristetraprolin (TTP, ZFP36) binds and destabilizes mRNA (R-MMU-450513)	1															■
Signaling by Hippo (R-MMU-2028269)	1															■
Branched-chain amino acid catabolism (R-MMU-70895)	1			■												
Citric acid cycle (TCA cycle) (R-MMU-71403)	1												■			
Sealing of the nuclear envelope (NE) by ESCRT-III (R-MMU-9668328)	1														■	
Signalling to ERKs (R-MMU-187687)	1															■
Azathioprine ADME (R-MMU-9748787)	1								■							
Carboxyterminal post-translational modifications of tubulin (R-MMU-8955332)	1														■	
RHO GTPases activate IQGAPs (R-MMU-5626467)	1														■	
Interconversion of nucleotide di- and triphosphates (R-MMU-499943)	1								■							
Pyruvate metabolism (R-MMU-70268)	1									■						
Glyoxylate metabolism and glycine degradation (R-MMU-389661)	1									■						
Gap junction assembly (R-MMU-190861)	1														■	
Gluconeogenesis (R-MMU-70263)	1						■									
Formation of the early elongation complex (R-MMU-113418)	1							■								
RHO GTPases activate WASPs and WAVEs (R-MMU-5663213)	1	■														
TBC/RABGAPs (R-MMU-8854214)	1											■				
TP53 regulates transcription of cell cycle genes (R-MMU-6791312)	1													■		
RAF activation (R-MMU-5673000)	1															■
ER-phagosome pathway (R-MMU-1236974)	1		■													
Recycling pathway of L1 (R-MMU-437239)	1														■	
Aggrephagy (R-MMU-9646399)	1														■	
Mitochondrial fatty acid beta-oxidation (R-MMU-77289)	1			■												
**TGF-beta receptor signaling activates SMADs (R-MMU-2173789)**	**1**				■											
EPHB-mediated forward signaling (R-MMU-3928662)	1	■														
MAP2K and MAPK activation (R-MMU-5674135)	1															■
Antigen presentation: folding, assembly and peptide loading of class I MHC (R-MMU-983170)	1		■													
Gap junction trafficking (R-MMU-190828)	1														■	
Negative regulation of MAPK pathway (R-MMU-5675221)	1															■
Gap junction trafficking and regulation (R-MMU-157858)	1														■	
TP53 regulates transcription of DNA repair genes (R-MMU-6796648)	1							■								
Signaling by retinoic acid (R-MMU-5362517)	1									■						
Retrograde transport at the Trans-Golgi-Network (R-MMU-6811440)	1											■				

UNCLASSIFIED: 7 — Cabp5 (▼), Calm2 (▲), Ceacam5 (▼), Csmd1 (▼), Hnrnpa2b1 (▲), Tmem191c (▲), Vnn3 (▲)

Exclusively differentially abundant in PCPNIC *vs.* CT

Eif4a1: Eukaryotic initiation factor 4A-I; **Fbxl16:** F-box/LRR-repeat protein 16; **Fkbp1a:** Peptidyl-prolyl cis-trans isomerase FKBP1A; **Gstm7:** Glutathione S-transferase Mu 7; **Psap:** Prosaposin; **Ptges3:** Prostaglandin E synthase 3; **Septin4:** Septin-4.

MALES

REACTOME PATHWAYS	# OF PTNS	GENES: Eif4a1	Fbxl16	Fkbp1a	Gstm7	Psap	Ptges3	Septin4
REGULATION: PCPNIC *vs.* CT		▲	▼	▼	▼	▲	▲	▼
**Immune system (R-MMU-168256)**	**4**	■	■	■		■		
**Metabolism (R-MMU-1430728)**	**3**				■	■	■	
**Signal transduction (R-MMU-162582)**	**3**			■		■	■	
Biological oxidations (R-MMU-211859)	2				■		■	
Metabolism of lipids (R-MMU-556833)	2					■	■	
**Adaptive immune system (R-MMU-1280218)**	**2**		■	■				
SMAC, XIAP-regulated apoptotic response (R-MMU-111469)	1							■
Release of apoptotic factors from the mitochondria (R-MMU-111457)	1							■
Aryl hydrocarbon receptor signalling (R-MMU-8937144)	1						■	
**Calcineurin activates NFAT (R-MMU-2025928)**	**1**			■				
HSF1 activation (R-MMU-3371511)	1						■	
Attenuation phase (R-MMU-3371568)	1						■	
Synthesis of prostaglandins (PG) and thromboxanes (TX) (R-MMU-2162123)	1						■	
Apoptotic factor-mediated response (R-MMU-111471)	1							■
Deadenylation of mRNA (R-MMU-429947)	1	■						
HSF1-dependent transactivation (R-MMU-3371571)	1						■	
**mTORC1-mediated signalling (R-MMU-166208)**	**1**			■				
ISG15 antiviral mechanism (R-MMU-1169408)	1	■						
Glycosphingolipid catabolism (R-MMU-9840310)	1					■		
**TGF-beta receptor signaling activates SMADs (R-MMU-2173789)**	**1**			■				
Glutathione conjugation (R-MMU-156590)	1				■			
**Intrinsic pathway for apoptosis (R-MMU-109606)**	**1**							■
**MTOR signalling (R-MMU-165159)**	**1**			■				
Glycosphingolipid metabolism (R-MMU-1660662)	1					■		
HSP90 chaperone cycle for steroid hormone receptors (SHR) in the presence of ligand (R-MMU-3371497)	1						■	
Deadenylation-dependent mRNA decay (R-MMU-429914)	1	■						
Ribosomal scanning anB37:C56d start codon recognition (R-MMU-72702)	1	■						
Translation initiation complex formation (R-MMU-72649)	1	■						
Activation of the mRNA upon binding of the cap-binding complex and eIFs, and subsequent binding to 43S (R-MMU-72662)	1	■						
Arachidonic acid metabolism (R-MMU-2142753)	1						■	
Ribosomal scanning anB37:C56d start codon recognition (R-MMU-72702)	1	■						
Translation initiation complex formation (R-MMU-72649)	1	■						
Activation of the mRNA upon binding of the cap-binding complex and eIFs, and subsequent binding to 43S (R-MMU-72662)	1	■						

Continues on the next page.

Exclusively differentially abundant in PCPNIC *vs.* CT

REACTOME PATHWAYS<br>**MALES** (continuation)	# OF PTNS	GENES: Eif4a1	Fbxl16	Fkbp1a	Gstm7	Psap	Ptges3	Septin4
**REGULATION: PCPNIC *vs.* CT**		▲	▼	▼	▼	▲	▲	▼
Arachidonic acid metabolism (R-MMU-2142753)	1						■	
Estrogen-dependent gene expression (R-MMU-9018519)	1						■	
Downstream signaling events of B Cell Receptor (BCR) (R-MMU-1168372)	1		■					
Signaling by TGF-beta receptor complex (R-MMU-170834)	1		■					
Cellular response to heat stress (R-MMU-3371556)	1						■	
Sphingolipid metabolism (R-MMU-428157)	1					■		
Antiviral mechanism by IFN-stimulated genes (R-MMU-1169410)	1	■						
**Apoptosis (R-MMU-109581)**	**1**							■
Phase II - conjugation of compounds (R-MMU-156580)	1				■			
Signaling by TGFB family members (R-MMU-9006936)	1		■					
L13a-mediated translational silencing of Ceruloplasmin expression (R-MMU-156827)	1	■						
GTP hydrolysis and joining of the 60S ribosomal subunit (R-MMU-72706)	1	■						
Phase I - functionalization of compounds (R-MMU-211945)	1						■	
Cap-dependent translation initiation (R-MMU-72737)	1	■						
Eukaryotic translation initiation (R-MMU-72613)	1	■						
Platelet degranulation (R-MMU-114608)	1					■		
Response to elevated platelet cytosolic Ca2+ (R-MMU-76005)	1					■		
ESR-mediated signaling (R-MMU-8939211)	1						■	
**Programmed cell death (R-MMU-5357801)**	**1**							■
Interferon signaling (R-MMU-913531)	1	■						

FIGURE S2a

Commonly differentially abundant in PCP *vs.* CT and PCPNIC *vs.* CT

Snca: Alpha-synuclein.

REACTOME PATHWAYS<br>**FEMALES**		# OF PTNS	GENE<br>Snca
**REGULATION:**	**PCPNIC *vs*. CT**		▲
	**PCP *vs*. CT**		▲
PKR-mediated signaling (R-MMU-9833482)		1	
Antiviral mechanism by IFN-stimulated genes (R-MMU-1169410)		1	
Interferon signaling (R-MMU-913531)		1	
Cytokine signaling in immune system (R-MMU-1280215)		1	

Commonly differentially abundant in PCP *vs.* CT and PCPNIC *vs.* CT

Stx1a: Syntaxin-1a.

REACTOME PATHWAYS<br>**MALES**	# OF PTNS	GENE<br>Stx1a
REGULATION: PCPNIC *vs*. CT		▼
PCP *vs*. CT		▼
LGI-ADAM interactions (R-MMU-5682910)	1	
Other interleukin signaling (R-MMU-449836)	1	
Insertion of tail-anchored proteins into the endoplasmic reticulum membrane (R-MMU-9609523)	1	
Acetylcholine neurotransmitter release cycle (R-MMU-264642)	1	
Norepinephrine neurotransmitter release cycle (R-MMU-181430)	1	
Serotonin neurotransmitter release cycle (R-MMU-181429)	1	
GABA synthesis, release, reuptake and degradation (R-MMU-888590)	1	
Dopamine neurotransmitter release cycle (R-MMU-212676)	1	
Glutamate neurotransmitter release cycle (R-MMU-210500)	1	
Neurotransmitter release cycle (R-MMU-112310)	1	
Protein localization (R-MMU-9609507)	1	
Transmission across chemical synapses (R-MMU-112315)	1	
Signaling by interleukins (R-MMU-449147)	1	
Neuronal system (R-MMU-112316)	1	
Cytokine signaling in immune system (R-MMU-1280215)	1	
Developmental biology (R-MMU-1266738)	1	

Commonly differentially abundant in NIC *vs.* CT and PCPNIC *vs.* CT

Atp1b1: Sodium/potassium-transporting ATPase subunit beta-1; **Cfl2:** Cofilin-2; **Eif4a1:** Eukaryotic initiation factor 4A-I; **Marcks:** Myristoylated alanine-rich C-kinase substrate; **Ptges3:** Prostaglandin E synthase 3.

REACTOME PATHWAYS<br>**FEMALES**	# OF PTNS	GENES<br>Atp1b1	Eif4a1	Marcks	Ptges3
**REGULATION:** PCPNIC *vs.* CT		▲	▼	▲	▼
NIC *vs.* CT		▲	▼	▲	▼
Aryl hydrocarbon receptor signalling (R-MMU-8937144)	1				■
HSF1 activation (R-MMU-3371511)	1				■
Acetylcholine regulates insulin secretion (R-MMU-399997)	1			■	
Attenuation phase (R-MMU-3371568)	1				■
Synthesis of prostaglandins (PG) and thromboxanes (TX) (R-MMU-2162123)	1				■
Deadenylation of mRNA (R-MMU-429947)	1		■		
HSF1-dependent transactivation (R-MMU-3371571)	1				■
Basigin interactions (R-MMU-210991)	1	■			
ISG15 antiviral mechanism (R-MMU-1169408)	1		■		
Ion homeostasis (R-MMU-5578775)	1	■			
HSP90 chaperone cycle for steroid hormone receptors (SHR) in the presence of ligand (R-MMU-3371497)	1				■
Deadenylation-dependent mRNA decay (R-MMU-429914)	1		■		
Ion transport by P-type ATPases (R-MMU-936837)	1	■			
Ribosomal scanning and start codon recognition (R-MMU-72702)	1		■		
Translation initiation complex formation (R-MMU-72649)	1		■		
Activation of the mRNA upon binding of the cap-binding complex and eIFs, and subsequent binding to 43S (R-MMU-72662)	1		■		
Arachidonic acid metabolism (R-MMU-2142753)	1				■
Regulation of insulin secretion (R-MMU-422356)	1			■	
Estrogen-dependent gene expression (R-MMU-9018519)	1				■
Integration of energy metabolism (R-MMU-163685)	1			■	
Cellular response to heat stress (R-MMU-3371556)	1				■
Antiviral mechanism by IFN-stimulated genes (R-MMU-1169410)	1		■		
Cardiac conduction (R-MMU-5576891)	1	■			
L13a-mediated translational silencing of Ceruloplasmin expression (R-MMU-156827)	1		■		
GTP hydrolysis and joining of the 60S ribosomal subunit (R-MMU-72706)	1		■		
Phase I - functionalization of compounds (R-MMU-211945)	1				■
**Cap-dependent translation initiation (R-MMU-72737)**	**1**		■		
**Eukaryotic translation initiation (R-MMU-72613)**	**1**		■		
ESR-mediated signaling (R-MMU-8939211)	1				■
Interferon signalin (R-MMU-913531)	1		■		
Muscle contraction (R-MMU-397014)	1	■			
Ion channel transport (R-MMU-983712)	1	■			
**Fatty acid metabolism (R-MMU-8978868)**	**1**				■
Cell surface interactions at the vascular wall (R-MMU-202733)	1	■			
Signaling by nuclear receptors (R-MMU-9006931)	1				■
Biological oxidations (R-MMU-211859)	1				■
**UNCLASSIFIED:**	1	Cfl2 (▼,▼)			

FIGURE S3b

Commonly differentially abundant in NIC *vs.* CT and PCPNIC *vs.* CT

Echs1: Enoyl-CoA hydratase_mitochondrial; **Eif2b5:** Translation initiation factor eIF-2B subunit epsilon.

**REACTOME PATHWAYS**	**# OF PTNS**	**GENES** Echs1	Eif2b5
**MALES**			
**REGULATION:** **PCPNIC *vs.* CT**		▼	▼
**NIC *vs.* CT**		▼	▼
Beta oxidation of butanoyl-CoA to acetyl-CoA (R-MMU-77352)	1		
Beta oxidation of hexanoyl-CoA to butanoyl-CoA (R-MMU-77350)	1		
Beta oxidation of octanoyl-CoA to hexanoyl-CoA (R-MMU-77348)	1		
Beta oxidation of lauroyl-CoA to decanoyl-CoA-CoA (R-MMU-77310)	1		
Beta oxidation of decanoyl-CoA to octanoyl-CoA-CoA (R-MMU-77346)	1		
Recycling of eIF2:GDP (R-MMU-72731)	1		
Mitochondrial fatty acid beta-oxidation of saturated fatty acids (R-MMU-77286)	1		
Branched-chain amino acid catabolism (R-MMU-70895)	1		
Mitochondrial fatty acid beta-oxidation (R-MMU-77289)	1		
**Cap-dependent translation initiation (R-MMU-72737)**	**1**		
**Eukaryotic translation initiation (R-MMU-72613)**	**1**		
**Fatty acid metabolism (R-MMU-8978868)**	**1**		
Translation (R-MMU-72766)	1		
Metabolism of amino acids and derivatives (R-MMU-71291)	1		

Commonly differentially abundant in all groups *vs.* CT

Arf5: ADP-ribosylation factor 5; **Gstm4:** Glutathione S-transferase Mu 4; **Ppp1cb:** Serine/threonine-protein phosphatase PP1-beta catalytic subunit; **Zic4:** Zinc finger protein ZIC 4.

REACTOME PATHWAYS	# OF PTNS	GENES: Arf5	Gstm4	Ppp1cb
**FEMALES**				
**REGULATION:** **PCPNIC *vs.* CT**		▲	▼	▼
**NIC *vs.* CT**		▲	▼	▼
**PCP *vs.* CT**		▲	▼	▼
Biosynthesis of maresin conjugates in tissue regeneration (R-MMU-9026762)	1		■	
Biosynthesis of DHA-derived sulfido conjugates (R-MMU-9026395)	1		■	
RHO GTPases activate PAKs (R-MMU-5627123)	1			■
RHO GTPases activate PKNs (R-MMU-5625740)	1			■
Biosynthesis of DHA-derived SPMs (R-MMU-9018677)	1		■	
Biosynthesis of specialized proresolving mediators (SPMs) (R-MMU-9018678)	1		■	
RAF activation (R-MMU-5673000)	1			■
Glutathione conjugation (R-MMU-156590)	1		■	
Regulation of PLK1 activity at G2/M transition (R-MMU-2565942)	1			■
COPI-dependent Golgi-to-ER retrograde traffic (R-MMU-6811434)	1	■		
COPI-mediated anterograde transport (R-MMU-6807878)	1	■		
Phase II - conjugation of compounds (R-MMU-156580)	1		■	
Golgi-to-ER retrograde transport (R-MMU-8856688)	1	■		
ER to Golgi anterograde transport (R-MMU-199977)	1	■		
G2/M transition (R-MMU-69275)	1			■
Transport to the Golgi and subsequent modification (R-MMU-948021)	1	■		
Mitotic G2-G2/M phases (R-MMU-453274)	1			■
Intra-Golgi and retrograde Golgi-to-ER traffic (R-MMU-6811442)	1	■		
Biological oxidations (R-MMU-211859)	1		■	
RHO GTPase effectors (R-MMU-195258)	1			■
Asparagine N-linked glycosylation (R-MMU-446203)	1	■		
RAF/MAP kinase cascade (R-MMU-5673001)	1			■
**UNCLASSIFIED:**	1	Zic4 (▼,▼,▼)		
